# Mesoporous Silica Nanoparticles: A Comprehensive Review on Synthesis and Recent Advances

**DOI:** 10.3390/pharmaceutics10030118

**Published:** 2018-08-06

**Authors:** Reema Narayan, Usha Y. Nayak, Ashok M. Raichur, Sanjay Garg

**Affiliations:** 1 Department of Pharmaceutics, Manipal College of Pharmaceutical Sciences, Manipal Academy of Higher Education, Manipal 576104, India; nsreema@gmail.com; 2 Department of Materials Engineering, Indian Institute of Science, Bengaluru 560012, India; amr@iisc.ac.in; 3 School of Pharmacy and Medical Science, University of South Australia, Adelaide, SA 5000, Australia; Sanjay.Garg@unisa.edu.au

**Keywords:** mesoporous silica nanoparticles, MCM-41, protocells, SBA-15, Stober’s synthesis, tetraethyl orthosilicate

## Abstract

Recent advancements in drug delivery technologies utilizing a variety of carriers have resulted in a path-breaking revolution in the approach towards diagnosis and therapy alike in the current times. Need for materials with high thermal, chemical and mechanical properties have led to the development of mesoporous silica nanoparticles (MSNs). These ordered porous materials have garnered immense attention as drug carriers owing to their distinctive features over the others. They can be synthesized using a relatively simple process, thus making it cost effective. Moreover, by controlling the parameters during the synthesis; the morphology, pore size and volume and particle size can be transformed accordingly. Over the last few years, a rapid increase in research on MSNs as drug carriers for the treatment of various diseases has been observed indicating its potential benefits in drug delivery. Their widespread application for the loading of small molecules as well as macromolecules such as proteins, siRNA and so forth, has made it a versatile carrier. In the recent times, researchers have sorted to several modifications in the framework of MSNs to explore its potential in drug resistant chemotherapy, antimicrobial therapy. In this review, we have discussed the synthesis of these multitalented nanoparticles and the factors influencing the size and morphology of this wonder carrier. The second part of this review emphasizes on the applications and the advances made in the MSNs to broaden the spectrum of its use especially in the field of biomedicine. We have also touched upon the lacunae in the thorough understanding of its interaction with a biological system which poses a major hurdle in the passage of this carrier to the clinical level. In the final part of this review, we have discussed some of the major patents filed in the field of MSNs for therapeutic purpose.

## 1. Introduction

Modern nanotechnology has evolved as the principal component of science in the current century. Over the years, diagnosis of diseases and its therapy is constantly leaping milestones due to the application of nanotechnology in the field of biomedicine. The evolution of nanomedicine and green technology for its production have been a great boon and have shifted paradigms in therapy and tissue engineering, owing to the advantages of nanocarriers such as a high surface area to volume ratio, unique features of surface modification and engineering to obtain particles of various sizes, shapes and different chemical characteristics. These have proven to be biocompatible, biodegradable and non-toxic which adds to its advantages [1,2,3,4,5]. Lipid-based nanocarriers [6,7,8], polymeric nanoparticles [9,10,11], dendrimers [12] have revolutionized the therapy for various conditions especially cancer and infectious diseases. Many of these products have been approved and are commercially available. Table 1 enlists some of the marketed nanomedicines.

Apart from the above mentioned organic nanoparticles, inorganic nanoparticles have also been widely explored for their application in biomedicine. Out of them, quantum dots, iron oxide nanoparticles have been approved and are commercially available. Carbon dots, nanoparticles of gold, silver, various other metal oxides, layered double hydroxide nanoparticles and silica nanoparticles have been widely used for various diagnostic and therapeutic purposes [2,18,19,20]. Of these, silica nanoparticles comprising of organic dyes and radioactive iodide known as Cornell dots (C dots) has successfully attained an important benchmark of safety by its approval for Phase I human trials which is vital for any substance requiring Investigational New Drug (IND) approval. C dots are core-shell silica nanoparticles containing fluorescent molecules within the silica core surrounded with silica shell which is further coated with polyethylene glycol (PEG). C dots were first developed by the Spencer T. Olin Professor of Engineering, Ulrich Wiesner from Department of Materials Science and Engineering at Cornell University [21,22].

Silica nanoparticles with mesopores–referred to as mesoporous silica nanoparticles (MSNs)–have gained wide popularity over the recent years. Its advantages of uniform and tunable pore size, easy independent functionalization of the surface, internal and external pores and the gating mechanism of the pore opening make it a distinctive and promising drug carrier. Scientists have successfully worked on the utilization of these carriers for loading variety of cargo ranging from drugs to macromolecules such as proteins [23,24], DNA [25,26] and RNA [27,28]. An exhaustive set of literatures are available and research is still underway in evaluating new avenues for the use of MSNs in drug delivery. Several reviews pertaining to MSNs in improving the solubility of the drug [29,30], as controlled/sustained drug delivery system [31], applications in biomedicine [32,33] have been published. The present review focuses on literatures published on a broad perspective of MSNs ranging from synthesis to the patents filed. In doing so, we realize that all the reported papers in each of the areas could not be discussed in detail. We have detailed and overviewed the recent research and patents applied for MSNs specifically on Mobil Crystalline Materials (MCM-41) and Santa Barbara Amorphous type material (SBA-15). An overview of the synthesis and theory behind the formation of MSNs is provided to discuss the factors affecting the shape and size of MSNs. The major research in the field of MSNs related to the biomedical applications for therapy based on small molecules and the related patents literature are included.

## 2. Origin of Mesoporous Silica Materials

**Figure 1:**
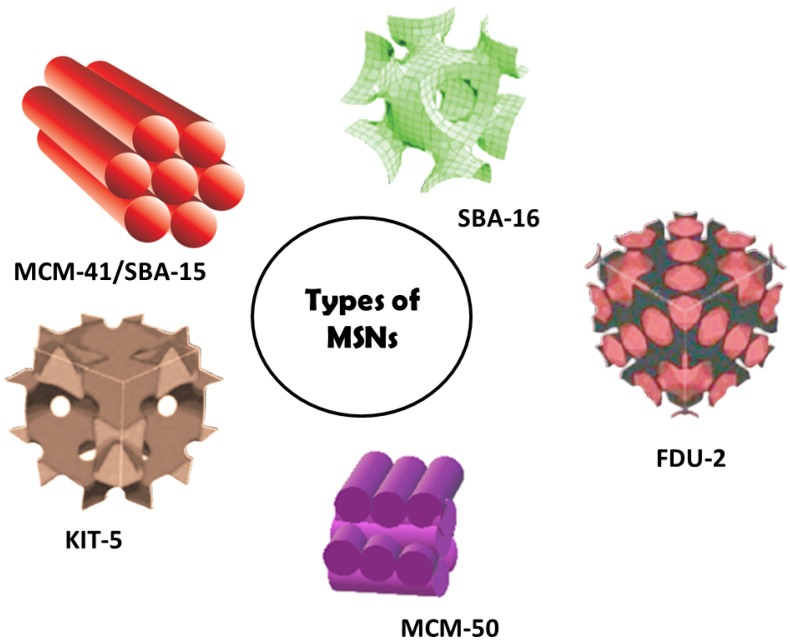
Representation of different types of mesoporous silica nanoparticles (MSNs).

Although the synthesis of mesoscopic materials dates back to 1970s, Mobil Research and Development Corporation was the first to synthesize mesoporous solids from aluminosilicate gels using liquid crystal template mechanism in the year 1992. They designated it as (Mobil Crystalline Materials or Mobil Composition of Matter) MCM-41. As per IUPAC, mesoporous materials are defined as the one having a pore size in the range of 2–50 nm and an ordered arrangement of pores giving an ordered structure to it [34,35,36]. The pore size of these could be varied and tuned through the choice of surfactants used. Generally, MCM-41 is hexagonal with a pore diameter of 2.5 to 6 nm wherein cationic surfactants were used as templates. MCM-41 is one of the most widely explored materials for drug delivery. Apart from this, various other materials of mesoporous nature have also been synthesized by varying the starting precursors and reaction conditions. These may vary in their structural arrangement or the pore size. MCM-48 has a cubic arrangement whereas MCM-50 has a lamella-like arrangement [37]. Non-ionic triblock copolymers like alkyl poly(ethylene oxide) (PEO) oligomeric surfactants and poly(alkylene oxide) block copolymers have also been used as a template which has been designated as SBA-11 (cubic), SBA-12 (3-*d* hexagonal), SBA-15 (hexagonal) and SBA-16 (cubic cage-structured) based on the symmetry of the mesoporous structure and the triblock polymers used. The ratio of ethylene oxide to propylene oxide was varied to achieve the desired symmetry of mesoporous materials. Highly ordered mesoporous structure of SBA-15 has also been widely used for the biomedical purpose. This was first synthesized by University of California, Santa Barbara and hence named Santa Barbara Amorphous type material (SBA). This is different from MCM in that they possess larger pores of 4.6–30 nm and thicker silica walls [38]. FSM-16, that is, folded sheets of mesoporous materials are another type of mesoporous materials, which are synthesized using quaternary ammonium surfactant as a template and layered polysilicate kanemite. Tozuka et al. demonstrated that FSM-16 could be used for pharmaceutical applications other than as an adsorbent and for catalysis [39]. Various other MSNs coined Technical Delft University (TUD-1), Hiroshima Mesoporous Material-33 (HMM-33), Centrum voor Oppervlaktechemie en Katalyse/Centre for Research Chemistry and Catalysis (COK-12) have been synthesized which vary in their pore symmetry and shape [40,41]. The structural characteristics of some mesoporous materials have been listed in Table 2. Figure 1 shows the representation of some MSNs. Of these, MCM-41, MCM-48, SBA-15, SBA-16 are widely employed for drug delivery. In addition, they have also been explored as adsorbents, catalysis and as biosensors. MCM-50, SBA-11 and SBA-12 have been reported to behave as excellent adsorbents and in catalysis.

## 3. Synthesis of MSNs

Stober was the pioneer in developing a system of chemical reactions for the synthesis of spherical monodisperse micron size silica particles [57]. From then on, the method is known as Stober synthesis. Many modifications have constantly been made to the Stober’s synthesis to yield monodisperse, ordered, nanosized silica particles. The synthesis of MSNs can be accomplished in basic, acidic and neutral conditions. Manipulating the reaction parameters resulted in particles with different shapes and sizes. The Stober’s method of synthesis was first modified by Grun et al. where they introduced a cationic surfactant as a template to yield a spherical rather than a hexagonal MCM-41 structure. They were successful in generating spherical MCM-41 with similar properties as that generated by other methods [58]. Constant research has led to a lot of variations in the synthesis conditions and methods to yield stable, monodisperse MSNs.

For MSNs to be an ideal carrier for drug delivery the particle size needs to be uniform; pore volume has to be large to enhance loading capacity. These parameters can be controlled during the synthesis by varying the pH of the reaction mixture, temperature, concentration of surfactant and silica source. The synthesis of MSNs occurs by liquid crystal template mechanism wherein hydrolysis and condensation of silica on the surface of surfactant micelles takes place. The liquid silica (tetraethyl orthosilicate) transforms to solid silica [59,60,61].

### 3.1. Mechanism of Formation of MSNs

**Figure 2:**
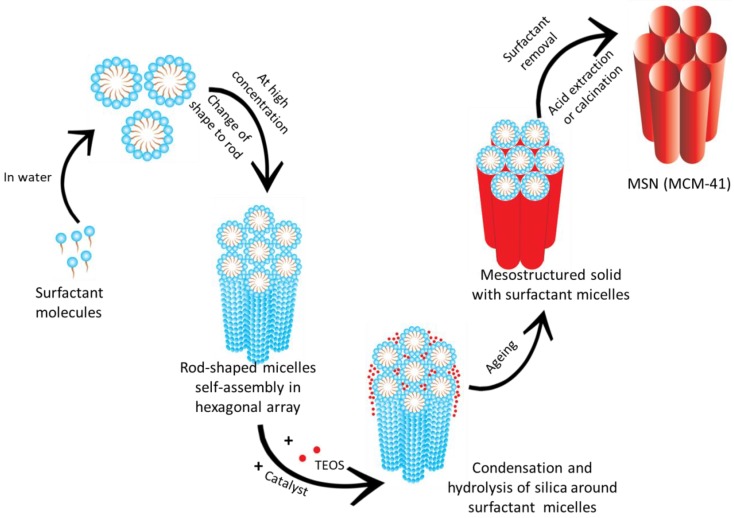
Mechanism of formation of Mobil Crystalline Materials No.41 (MCM-41).

A thorough understanding of the mechanism of formation of MSNs is essential to obtain particles with desired properties for drug delivery. The early reports on the mechanism suggested that the silica network gets built throughout the liquid–crystalline phases of non-ionic surfactants. This is particularly true for materials prepared from a dilute solution of surfactants as no evidence of regular mesostructured materials was observed [62]. The literature has shown that either the hydrolysed silica gets adsorbed around the micelles or in the case of SBA-15, the surfactant and the silica interact at the initial stage and form a core shell-like structure [63]. The mechanism for the formation of MCM-41 is represented in Figure 2. Efforts have been on since then by research groups to unveil the exact mechanism behind the formation of MSNs.

The in-situ usage of time-resolved small-angle neutron scattering (SANS) has been used to study the formation of MSNs. Using this method, they were able to predict the changes happening concurrently with the formation process. It was observed that during the early hydrolysis (~40 s) of silica source tetramethyl orthosilicate (TMOS), the silicate ions tend to adsorb around the surfactant micelles during the growth phase. As the charge around the surfactant reduces due to the initial hydrolysis and the condensation of the silica precursor, the intermicellar repulsion reduces, allowing the further formation of small aggregates of silica. After ~400 s, the reaction mixture contained sufficiently discrete hexagonally ordered mesopores of silica which was confirmed by transmission electron microscopy (TEM) studies. This is in accordance with the previously proposed ‘current bun model’ for the mechanism of formation of MSNs [64,65].

Another mechanism named ‘swelling-shrinking mechanism’ was proposed for the formation of MSNs utilizing the technique of time-resolved synchrotron small-angle X-ray scattering (SAXS). This mechanism holds well when tetraethyl orthosilicate (TEOS) alone is used as the precursor in the absence of any other solvent like ethanol. TEOS being oil-like monomer showed phase separation under static condition, whereas, under vigorous stirring, an emulsion-like system was obtained. Initially, cetyltrimethylammonium bromide (CTAB) forms ellipsoidal micelles with an inner core consisting of the hydrophobic tail. When TEOS is added, it gets solubilized in the hydrophobic core, thus enlarging the micelles and resulting in the transformation of micelle shape from ellipsoidal to spherical. On hydrolysis of TEOS, the monomers become hydrophilic and are released into the aqueous surroundings. The negatively charged hydrolysed monomers of TEOS get adsorbed onto positively charged CTAB micelles via electrostatic attraction. On complete consumption of the TEOS within the hydrophobic core, the micelles shrink and become smaller in size. As this process of hydrolysis and condensation occurs simultaneously, the micelles shrink continuously until all the TEOS gets hydrolysed and form silica shell around the micelles. The neighbouring micelles aggregate, resulting in particle growth forming a mesoporous structure [66].

### 3.2. Approaches for the Synthesis of MSNs

Majority of the MSNs are fabricated by modified Stober’s method otherwise popularly known as a sol-gel process. Sol-gel chemistry is a widely explored process for the synthesis of many inorganic materials. It involves the hydrolysis and condensation of the alkoxide monomers into a colloidal solution (sol), which acts as a precursor to form an ordered network (gel) of polymer or discrete particles. A typical sol-gel process takes place in the presence of an acid or a base catalyst. Depending on the reaction conditions and the molar ratio of Si/H_2_O, the alkoxide group gets hydrolysed. The rate of hydrolysis proceeds faster in basic conditions compared to acidic. Condensation succeeds the hydrolysis step and the effective condensation depends on the hydrolysis step. Multiple condensation results in a chain-like structure in the sol and network-like structure in gel form [67]. The schematic representation of the reaction is shown in Equation (1).

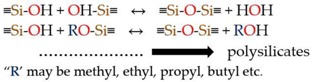
(1)

To yield particles of the desired size and to enhance the properties of MSNs, the sol-gel process has been modified. Some of the approaches are discussed below:

A simple quenching approach was adopted by Mann et al. to synthesize small sized and ordered MSNs. The reaction was quenched after 40 s by the addition of an excess of water followed by neutralization to pH 7 with dilute hydrochloric acid after a delay time ranging from 60 s to 220 s. The rest of the experimental condition and reagents were maintained the same as in the routine procedure. It was observed that dilution reduces coalescence, and neutralization reduces the rate of silica condensation. The results showed that greater the delay in the neutralization, larger the particle size [68,69]. Similar results were observed by Moller group. The colloidal suspension obtained was found to be stable for long periods of time. However, the MSNs produced by this technique were found to be less ordered in a structure which could be due to scale-up issues during dilution [70].

Evaporation-induced self-assembly (EISA) is another approach for the synthesis of MSNs. In this technique, all the reactants undergo concentration changes during evaporation throughout the process. This results in the organization of a liquid-crystal like template of the silica precursor. In this method, the required concentration of precursor formulations was prepared in ethanol/water solvent with a surfactant. This was converted to monodisperse droplets via injection into an aerosol generator. The droplet size can be controlled by altering its orifice. The alcohol evaporation during drying induces micelle formation and the co-assembly of silica-surfactant into liquid-crystal mesophases [71]. Fontecave et al. modified the EISA method by incorporating different amphiphilic drugs (stearoyl choline, sophorolipid and glucosyl-resveratrol) which behave both as a structure directing agent as well as an active cargo. Sol compositions containing TEOS, drug, water, ethanol and HCl were prepared and injected into an aerosol apparatus. A spray dryer with sufficient air flow and pressure was provided to convert the droplets into solid particles. Characterization of the particles for its pore size and structure using TEM revealed similar results to those with CTAB as surfactant template. Even though the mesostructure formation was not that good, the drug loading was found to be high in all the three cases with complete elimination of burst release. This method would be an ideal strategy for hydrophilic drugs functionalized with hydrophobic tails. Due to the absence of the surfactant template, intrinsic toxicity can be reduced [72].

A recent widely used modification to the synthesis of parent MSNs is encapsulating drugs in hollow mesoporous silica nanoparticles (HMSNs). Their large hollow cavity inside each MSN has garnered tremendous attention. These hollow cavities are capable of holding a high amount of drug compared to its non-hollow counterparts. This unique property of HMSNs makes it widely useful in cancer therapy and imaging [73,74]. Shi et al. were one of the first groups to report the synthesis of HMSNs [75,76]. Preliminary studies to ascertain its enhanced loading capacity was performed using ibuprofen as the model drug wherein, HMSNs showed an enhanced drug loading of 744.5 mg/g, as compared to 358.6 mg/g of MCM-41 [75]. The frequently used method for the synthesis of HMSNs includes the ‘core-templating method’. In this approach, many soft/hard templates are used to form the core followed by coating with desired substance at different concentrations to obtain a shell around the substrate with a desired thickness. Subsequently, the core template can be eliminated by calcination or treatment with a suitable solvent leaving behind the shell with a hollow core [77]. She et al. synthesized HMSNs using eudragit S-100 and Triton X-100 as the core-shell template [78]. HMSNs using SiO_2_@CTAB-SiO_2_ nanoparticles as templates were synthesized by selective etching process [79]. Ghasemi et al. synthesized HMSNs using poly tert-butyl acrylate (PtBA) nanospheres as core-forming hard templates in the presence of CTAB as soft templates [80].

Many hard/soft templates like vesicles, polymeric micelles [81,82], gold [83] or silica nanoparticles were used to construct templates [84,85]. Lin and collaborators proposed a new technique for the synthesis of MSNs using water-in-oil microemulsion as a template. The advantages of this method were the uniformly sized particles obtained compared to other methods. Also, the microemulsion is said to be thermodynamically stable [86].

As the commonly used sol-gel process is a laborious, time consuming process, many different fast methods were used for the synthesis of MSNs. Ling and Su proposed a low-cost electrochemistry assisted approach to synthesize MSNs in large-scale. The formation of MSNs was accomplished by the production of hydroxide at the stainless steel substrate/solution interface which resulted in the self-assembly of surfactant micelles and the polycondensation of silica precursors [87]. Microwave assisted technique for the synthesis of MSNs could be another low-cost approach for the synthesis of MSNs. Various reports show that MSNs with ordered pore size and arrangement could be rapidly synthesized by this method [88,89]. Another rapid, cost effective method for the fabrication of MSNs is sonochemcial synthesis. The use of photoacoustic cavitations during the process was found to generate ordered MSNs, giving scope for the fine tuning of the process in a shorter duration of time [90,91].

### 3.3. Raw Materials Used and Factors Affecting the Characteristics of MSNs

The three main elements that form the heart of MSN includes a silica precursor (tetraethyl orthosilicate-TEOS, tetramethyl orthosilicate-TMOS, tetramethoxyvinylsilane- TMVS, sodium meta-silicate and tetrakis(2-hydroxyethyl) orthosilicate- THEOS), a surfactant (non-ionic or cationic surfactant) as a structure directing agent (SDA) and a catalyst. Other additives like cosolvents, compounds to prevent aggregation may also be incorporated based on the requirements. In order to ensure the scale-up of MSNs at reasonable cost, natural perlite materials like pumice rock, rice husk, and renewable biomass could also be explored for the synthesis of MSNs [92,93]. The common chemical constituents explored so far are listed in Table 3.

The particle size, pore size and morphology of MSNs can be successfully modulated as required by varying the reaction conditions (relative amounts of alkoxysilane, water, catalyst) and temperature.

#### 3.3.1. Control of Particle Size

Particle size is a very important feature for the biomedical application of MSNs as drug carriers. Hence careful tuning of particle size is essential for effective drug delivery. pH of the reaction medium plays a pivotal role in governing the size of MSNs. The particle size can be effectively controlled by adding suitable additive agents like alcohols, amine, inorganic bases and inorganic salts. These agents alter the hydrolysis and condensation of silica precursor. They accelerate the reaction kinetics thus resulting in particles of smaller size. Moller et al. replaced the often used base catalyst sodium hydroxide (NaOH) and ammonium hydroxide (NH_4_OH) with triethanolamine (TEA). In addition to conferring a basic pH, it also acts a complexing agent to obtain discrete nanoparticles. When the molar ratio of TEOS: TEA was changed from 1:1 to 1:4, the largest particle size was observed with the ratio 1:4 [97]. Qiao [96] suggested that the initial pH value of the system greatly affects the particle size of MSNs. When they provided Na_2_HPO_4_-NaH_2_PO_4_ as a source of OH^−^ ions, particle size was found to increase with an increase in the initial pH of the solution. El-Toni et al. observed that on increasing the concentration of ammonia beyond a certain level, the agglomeration of silica particles takes place due to the increase in the ionic strength of the reaction medium [113]. Bouchoucha et al. also reported the efficiency of TEA in producing well-dispersed nanoparticles [114]. Use of l-lysine as a base catalyst was found to hinder the growth of silica particles, thus resulting in sub-nanometre size particles. This may be due to the electrostatic interaction between the protonated amine groups of l-lysine and a deprotonated hydroxyl group on silica surface further delaying the condensation process [115]. PEG-silane capping on the surface of silica particles was also found to effectively attenuate the particle growth process by steric stabilization. When PEG-silane was added immediately after addition of TMOS, the particle size was found to be 5 nm but when it was added at a delay of 50 min after addition of TMOS, the particle diameter increased to >13 nm [115]. A change from monodisperse to heterogeneous particle size distribution was observed when the amount of silica precursor TEOS was increased, which may be attributed to the secondary condensation reactions taking place due to the presence of excess silica precursor which starts producing new nuclei amongst the already existing silica particles [107,116]. A similar trend of results was obtained by Chiang et al. who found that particle size was found to increase with an increase in the amount of TEOS [117]. An increase in the particle size was observed by using different tetraalkoxysilane with different alkoxy groups (Si(OR)_4_, R = Me, Et, Pr and Bu). Along with this, the addition of alcohols also influenced the particle size of the MSNs. This may be due to the alteration in the hydrolysis rate [118]. Low concentration of CTAB as surfactant yielded a homogenous spherical particle size distribution. On investigating the effect of CTAB on the particle size, it was observed that transformation from discrete spherical to agglomerates was observed due to variation in the hydrolysis and micellization of CTAB [119]. By tuning the concentration of F127 polymer, the particle size could be controlled. An increase of particle size up to 300 nm was reported with an increase in the triblock copolymer Pluronic F127 concentration. Reports state that a balance between the molar composition of various reactants was necessary to obtain MSNs with desired size and quality [120]. The reaction parameters equally influenced the mean particle size of MSNs. On increasing the temperature of the reaction from 30 to 70 °C, an increase in the particle size from 28.91 nm to 113.22 nm was observed. This may be due to the increase in the rate of reaction leading to polycondensation of the silica monomers resulting in a dense silica structure and a larger size [107].

#### 3.3.2. Control of Pore Size, Pore Volume and Mesostructural Ordering

Depending on the type of surfactant, the pore size of MSNs can be varied. The longer chain length of surfactant results in MSNs with larger pores and those with short chain length gives MSNs with smaller pores [121,122,123,124]. The concentration of TEOS influenced the mesostructural ordering of the particles. The higher amount of TEOS showed a disordered mesostructure whereas lesser amount was not sufficient to form a mesoporous structure [117]. The concentration of surfactant CTAB was also found to have a profound impact on the mesostructural arrangement of the particles. A lower concentration of surfactant fails to form micelles and hence the resulting nanoparticles will be template deficient whereas the too high concentration of CTAB may result in a disordered structure [35]. Hence an optimum balance has to be struck between various reagents used. Moreover, mesostructural ordering takes place in dilute aqueous solutions [125]. Addition of N, *N*-dimethylhexadecylamine (DMHA) behaves as a pore size mediator and thus helps with efficient control of pore size as per requirements [126]. Pore size was also found to have a profound influence on the release rate of the drugs as observed using ibuprofen [127,128]. The selection of surfactant species greatly influences the mesostructural ordering of the nanoparticles and the pore size. Effect of the templating agent due to change in the counterions present in cetyltrimethylammonium was studied. Cetyltrimethylammonium chloride (CTAC) as pore generating template produced MSNs with the wormhole-like arrangement. On changing the counter ion to a much larger tosylate ion (CTATOS), the pore radius was found to increase and the pore morphology changed from wormlike to stellate [129]. Of late, novel strategies have been adopted by scientists to bring about modifications in the properties of the MSNs in order to overcome the drawbacks of the traditional MSNs such as small pore size and poor particle size uniformity. In this regard, Huang et al. [130], synthesized highly monodisperse silica nanoparticles having a large pore size with dendritic morphology using a novel dual templating sol-gel reaction by mixing partially fluorinated short chain anionic fluorocarbon surfactant, Capstone FS-66 and CTAB. An interesting observation was made by them showing that an increase in the amount of Capstone FS-66 incorporated resulted in a change in the morphology. The particle size was larger with a dendritic channel pore structure. As the amount of Capstone is further increased, the morphology transforms into a flower-like large dendritic structure. The same strategy was used by Yu et al., who synthesized dendritic MSNs with particle size of less than 200 nm using imidazolium ionic liquids with different alkyl lengths as cosurfactants and Pluronic F127 as a particle growth inhibitor. They observed that neither the reaction temperature nor the time showed any influence on the particle size of MSNs [131].

#### 3.3.3. Control of Shape

The shape of the MSNs greatly affects the cellular uptake and biodistribution of MSNs. Hence stringent control of the shape of MSNs is important to regulate its excretion and other effects in vivo [132]. A clear picture of the relationship between particle shape and cellular responses was demonstrated in the paper by Huang et al. [133]. Till date, spherical MSNs have been widely explored for their drug delivery potential. However, the non-spherical MSNs are seldom used. By carefully controlling the reaction conditions, non-spherical materials with rod, ellipsoid, film, platelet, sheet and cube shapes could be generated. The molar concentration of surfactant, water, base catalyst and TEOS was found to have an impact on the morphology of the MSNs. Cai et al. generated MSNs with different shapes like spherical, silica rods and micrometre-sized oblate silica by manipulating the concentration of TEOS, NaOH/NH_4_OH and CTAB [134]. By regulating the amount of dodecanol as a soft template and the temperature of synthesis, a broad range of silica particles could be realized varying from sphere to shell, rugby, peanut, hollow and yolk shell-like structures. The six different particles fabricated had controlled size, porosity, interior spaces and shell structure. Their results showed that inclusion of dodecanol as a soft template resulted in particles with different morphologies [135]. This indicates that any changes in the micelle structure at the initial stage leads to a change in the particle morphologies. MSNs of various shapes can be obtained with a wide range of aspect-ratio. Rod-shaped MSNs are widely exploited counterparts of spherical MSNs. These can be obtained by varying the reaction parameters in a typical sol-gel reaction. An increase in the amount of catalyst and addition of co-solvent like heptane, change in temperature, varying the molar composition of reactants yield rod-shaped MSNs [133,136,137]. Ellipsoidal shaped MSNs also form a part of drug delivery carrier. Inability to retain its shape due to minimization of surface free energy leading to spherical particles poses a major challenge. These MSNs can be synthesized by the introduction of a co-surfactant [138], the addition of potassium chloride and ethanol [139]. Platelet-shaped MSNs with high pore accessibility can be synthesized by addition of low amounts of ammonium fluoride and heptane [137], the presence of non-ionic block copolymer P104 [140], using a ternary surfactant system of cetyltrimethylammonium bromide–sodium dodecyl sulfate–Pluronic123 [141].

Apart from the silica precursors, certain organosilanes were incorporated, which performed the dual function of shape transformation and surface functionalization. Morphological variants of MSNs could be synthesized by the co-condensation method of incorporation of organosilanes. The particle morphology depends on the type and amount of the organoalkoxysilane precursors introduced [36,142]. Various shapes of MSNs such as spheres, rods and hexagonal tubes could be generated using 3-aminopropyltrimethoxysilane (APTMS), *N*-(2-aminoethyl)-3-aminopropyltrimethoxysilane (AAPTMS), 3-[2-(2-aminoethyl amino) ethylamino] propyltrimethoxysilane (AEPTMS), ureidopropyltrimethoxysilane (UDPTMS), 3-isocyanatopropyltriethoxysilane (ICPTES), 3-cyanopropyltriethoxysilane (CPTES) and allyltrimethoxysilane (ALTMS) as organoalkoxysilanes. The transformation in shape of the MSNs may be attributed to the different types of interaction such as hydrogen bonding, hydrophobic interactions between the organoalkoxysilane and the surfactant template.

## 4. Drug Loading and Release of Drugs from MSNs

The unique feature of MSNs which makes it a widely exploited carrier for drug delivery is its high loading capacity due to the large pore volume and surface engineering properties both on the external and internal surface for better drug targeting.

### 4.1. Drug Loading

The drug loading is mainly based on the adsorptive properties of MSNs. Both hydrophilic and hydrophobic cargos can be incorporated into the pores of MSNs. Owing to their large pore volume, MSNs inherently possess greater loading capacity compared to other carriers. Nevertheless, extensive work has been carried out to further enhance the loading of the drugs. Synthesis of HMSNs is one such approach to enhance the loading of MSNs (explained in Section 3.2). She et al. attempted to increase the loading of 5-fluorouracil (5-FU) into hollow MSNs by functionalizing the surface silanol groups with different silanes *viz*, octadecyltrimethoxysilane (OTMS), (3-aminopropyl) triethoxysilane (APTES), 3-cyanopropyltriethoxysilane (CPTES). An improved loading of 28.89% was observed for amine functionalized HMSNs compared to plain HMSNs with 18.34%. This may be via the electrostatic interactions between the negatively charged 5-FU and positively charged amino modified HMSNs. A similar strategy could be used for improving the loading capacity of drugs by electrostatic attractions by varying the type of functionalization [78]. However, a contrasting theory was put forth by Wang et al. whose paper suggested that loading of a drug prior to surface grafting yielded a carrier with high loading as compared to grafting followed by loading of the drug [143,144]. Compared to MSNs, HMSNs proved to be a better carrier in terms of loading capacity due to their hollow cavities. 3–15 times higher loading of drugs was observed in HMSNs when compared to MSNs. In addition, dual loading of drugs was also achieved using the same carrier [74,75,145]. The loading capacity of MSNs could be further enhanced by utilizing polymer gatekeeping for the entrapment of hydrophobic drugs [146]. Consecutive drug loading process which increases the intermolecular interactions can also lead to improved loading of the drugs [147]. An increase in the drug feeding ratio was also found to have a profound influence on the loading capacity of MSNs [145,148]. The pore volume of MSNs is the major factor which dictates the loading of the drug. Hence pore expansion strategy can be adopted to introduce and hold a large amount of cargo. Pore swelling agents such as alkanes/ethanol, triisopropyl benzene (TIPB), trioctylamine (TOA), decane and *N*,*N*-dimethylhexadecylamine (DMHA) aid pore expansion [129,149,150]. Table 4 gives a comparison of the drug loading capacity of various MSNs.

### 4.2. Release of Drugs from MSNs

The release profile of drugs from MSNs mainly depends on its diffusion from the pores which can be tailored by modifying the surface of the MSNs to suit the biological needs. The decisive factor responsible for controlling the release is the interaction between the surface groups on pores and the drug molecule [154]. It was observed that drug loading followed by surface functionalization with amine groups played a significant role in sustaining the drug release as compared to the systems which were functionalized first and then loaded with the drug. This could be attributed to the loading of the drugs within the pores and the capping with APTES which prevents the drug release. If the surface was first functionalized and then loaded, there are possibilities that the drug will get adsorbed on the surface of MSNs resulting in burst release. The role of APTES concentration in drug release was studied, the results of which revealed that a change in the APTES concentration played a vital role in controlling the drug release from the pores [143]. Aspirin loading and release from the MSNs were studied by post-synthetic grafting as well as co-condensation method. It was observed that co-condensation method showed a greater drug loading compared to the other method. In case of plain MCM-41, weak interaction between aspirin and silanol groups resulted in faster drug release following Fick’s diffusion. With amino functionalized MCM-41, the strong interaction between the amine group and aspirin resulted in slow drug release especially for the co-condensed MCM-41 [155]. Echoing similar results, the release of ibuprofen from SBA-15 was found to be greatly influenced by the surface modification. In case of amino-functionalized SBA-15 by one-pot synthesis, complete drug release was observed at the end of 10 h whereas the release from that of post-synthetically modified SBA-15, the release of ibuprofen up to 3 days was observed [156]. Table 5 presents the comparison of release rates of different MSNs.

## 5. Applications of MSNs in Drug Delivery

**Figure 3:**
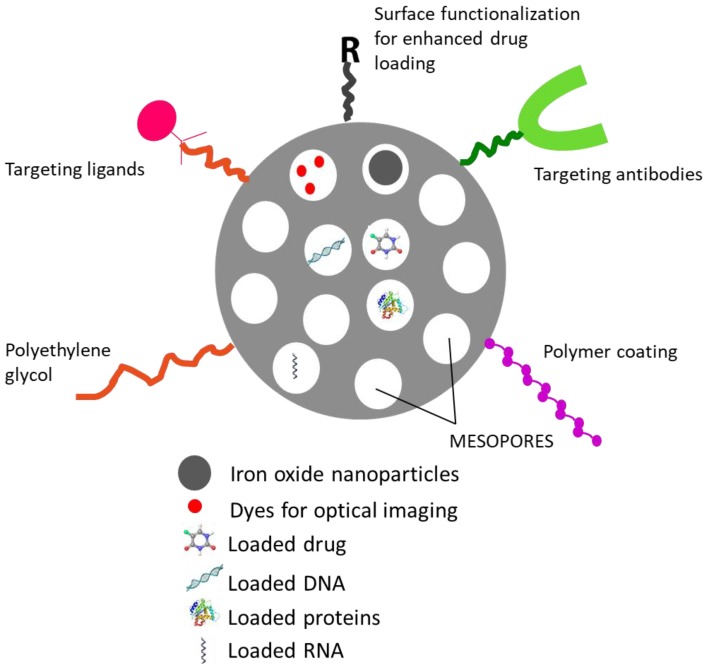
Illustration of versatility of MSN as a carrier in loading variety of drugs.

MSNs have been widely utilized for a variety of purposes ranging from its use in medicine, as a catalyst in chemical synthesis, adsorbents to adsorb wastes, toxic substances and also as sensors. One of the advantages of MSNs is the ease with which it can be functionalized based on the requirements for a wide variety of applications to control the release of drugs. Figure 3 shows a pictorial representation depicting the versatility of MSN carrier. Vast research is being conducted in utilizing these carriers for drug delivery. In the following section, the major applications of MSNs for drug delivery are discussed. Table 6 provides a list of few of the diseases for which MSNs have been exploited.

### 5.1. Targeted Antitumor Therapy Using MSNs

The property of surface functionalization of MSNs is widely explored to enhance the site-specific delivery of drugs and avoid side effects. It is a versatile carrier where drugs with different physicochemical properties can be loaded and further functionalized for effective therapy. They can accumulate significantly in the tumour due to the enhanced permeation and retention (EPR) effect [179]. Active targeting aids in maximizing the uptake of actives into the cells. Based on the difference between the normal and tumour cells, suitable receptors overexpressed on tumour cells are selected and ligands specific to those receptors are conjugated on the surface of the MSNs. Specific drug delivery can be achieved by anchoring MSNs with targeting ligands. Folic acid (FA) is a well-known ligand which complements the folate receptors overexpressed on tumour cells. The FA conjugation on the surface of MSNs is brought about by an amide linkage between the carboxyl group of FA and amine group of aminopropyltriethoxysilane (APTES). Ma et al. [180] delivered 5-aminolevulinic acid for photodynamic therapy by conjugating folic acid on the surface of HMSNs against B16F 10 skin cancer cells. The developed formulation was observed to show a high photocytotoxicity to the cancer cells. Similar reports were presented wherein the presence of amine functionalization on the surface aids in the binding of folic acid covalently on the surface of the receptors to ensure selective uptake of doxorubicin (DOX) in breast cancer cells. The apoptosis and cellular uptake studies revealed that FA-MSN-NH_2_-DOX showed higher internalization into the cells as compared to MSN-NH_2_-DOX [181]. Hyaluronic acid (HA) is another widely explored ligand for targeting CD44 receptors overexpressed on cancer cells. Zhang et al. reported an HA functionalized DOX-MSN which behaved both as enzyme responsive and receptor-mediated delivery system. The increased uptake by colon cancer cells and low in vivo toxicity to the body was proved by in vivo tumour growth inhibition and biodistribution studies. In addition to receptor-mediated uptake, the release of DOX was triggered by hyaluronidase enzyme present in tumour microenvironment [182]. Similar results were reported by Gary-Bobo et al. [183] who fabricated HA-MSNs for photodynamic therapy against colon cancer. The experiments were conducted on HCT-116 colon cancer cell lines which showed the higher efficiency of HA-MSNs owing to their CD44 receptor targeted action as compared to that of plain MSNs. Another novel approach for active targeting of hepatoma cells was reported wherein lactosaminated (Lac) MSNs were designed. These novel delivery systems showed a promising outcome as it had the ability to be endocytosed by asialoglycoprotein (ASGPR) receptors present on the surface of hepatocytes. The findings were supported by the results from cellular uptake studies in ASGPR-positive cells (HepG2 and SMMC7721) wherein Lac-MSNs showed improved cellular uptake when compared to that of plain MSNs. The results also highlighted an interesting point wherein the ligand lactose was recognized only when conjugated with MSNs and not in its free form [184]. Analogues for targeting mannose-6-phosphate receptor overexpressed in cancer cells were grafted onto MSNs to enhance the uptake by tumour cells. These were found to show positive results for the treatment of prostate and colon cancers [185,186]. Arginine-glycine-aspartic acid (RGD) was demonstrated to be selectively engulfed by ανβ3 and ανβ5 integrin receptors, which are overexpressed in diverse tumours. The drug-loaded surface engineered RGD-MSNs were thwarted by the normal cells and taken up by the liver cancer cells thus improving the treatment efficacy [187]. In addition to molecular targeting, efforts were also made to club positron-emission tomography (PET) imaging and chemotherapy into a single carrier. Sunitinib as a model anticancer drug was loaded onto the MSNs and surface engineered with cyclo-(Arg-Gly-Asp-D-Tyr-Lys) peptide (cRGDyK) and polyethylene glycol. The efficacy of the receptor uptake was confirmed by flow cytometry studies, PET imaging and histopathological studies. The studies were carried out in U87MG human glioblastoma cells and athymic nude mice were used for in vivo experiments. The tumour uptake of the nanoconjugates was found to be lower in the case of plain HMSNs as compared to that of cRGDyK conjugated HMSNs [188]. Numerous positive outcomes have been demonstrated using a plethora of ligands which are grafted onto the surface of MSNs rendering it target specific. Table 7 lists a few of the ligands which have been explored for cancer drug delivery using MSNs.

The surface functionalization of MSNs is not limited to just one ligand. Multiple ligands can be anchored onto the surface of MSNs for receptor targeting. For example, the surface of MSNs loaded with chlorambucil was functionalized with HA and RGD peptide for a synergistic effect. The in vitro cell line studies on human ovarian cancer (SKOV-3) cells showed a significant improvement in the uptake of the MSNs by CD44 and integrin receptors as compared to that of single ligand or plain MSNs [197].

In the recent times, a novel chemotherapeutic strategy utilizing copper impregnated MSNs (Cu-MSNs) have been explored for their potential reactive oxygen species (ROS) mediated apoptosis of cancer cells. Kankala et al. developed a novel Cu-MSN loaded with a catalase inhibitor, 3-amino-1,2,4-triazole (AT) for ROS mediated killing of cancer cells. On uptake by cells and delivery to the endosomal compartment (pH 5.0), the binding between copper and AT breaks off which releases AT to inhibit catalase activity. This in turn, leads to an increase in the ROS production induced by catalysis of copper on MSNs which aids in the cancer cell apoptosis. Positive results were obtained by them when evaluated in HT-29 cells [198]. An effort to overcome the multidrug resistance observed in chemotherapy was made utilizing Cu-MSNs loaded with DOX as model drug. In this system, DOX was conjugated to copper metal through a pH sensitive coordination link susceptible to acidic tumour environment (pH 5.0–6.0). This entire system was further coated with liposomes comprising of cholesterol, d-α-tocopheryl polyethylene glycol 1000 succinate (TPGS), MPEG-2000-DSPE (1,2-Distearoyl-phosphatidylethanolamine-methyl-polyethyleneglycol conjugate-2000), a P-gp inhibitor. Copper ions play a synergistic role in enhancing the intracellular ROS levels which results in killing of cancer cells efficiently. Positive results were obtained by the group when the delivery system was tested in DOX-resistant tumour (MES-SA-DX-5 derived from human uterine sarcoma) and HT-29 cell lines. The antitumor activity of Liposomes-Cu-MSN-DOX was found to be higher when compared to that of pure DOX. The antiproliferative activity of the optimized formulation was found to be profoundly higher in drug resistant cells than the sensitive cells [199].

### 5.2. MSNs for Anti-Inflammatory Activities

The unique properties of MSNs have been used to accommodate various anti-inflammatory drugs and control their release rate. MCM-41 and SBA-15 are the two widely used silica drug carriers. Ibuprofen was loaded onto multimodal pore channels and the effect on its release was studied. On analysing the release data, it was observed that the release of the drug occurs in three stages *viz*, initial rapid release of the drug adsorbed on the surface of MSNs followed by prolonged release of the drug entrapped in the small pores present in the periphery of the particles having a different pore structural orientation compared to the inner mesopores. The final prolonged release stage occurs by the drug embedded deep in the long length pores of the particles [200]. The bulk of the research work on MSNs for effective delivery of anti-inflammatory drugs has been carried out using ibuprofen as a model drug. Efforts were made to modulate the release rate of the drug by various organic modifications of the surface silanol groups. Surface functionalization was found to alter and also stabilize the drug release rates [127,201]. SBA-15 has also been used to precisely deliver ibuprofen and other anti-inflammatory agents. One such work by Ahmadi et al. demonstrated a change in the release rate of ibuprofen when the surface of the carrier was modified with aminopropyl groups. Plain SBA-15 was not able to sustain the release of the drug due to weak interaction between the surface silanol group and the carboxyl group of ibuprofen. However, on amino functionalization, a relatively prolonged release of the drug was observed due to the strong interaction between the amino groups of silica surface and carboxyl groups of ibuprofen [202]. Other similar studies were carried out to successfully deliver aspirin [155] and indomethacin [203] using MSNs with high drug loading and slow release profile.

### 5.3. Gated Drug Release/Controlled Drug Delivery

Although the length and the pore structure altered the drug release rate, efforts are constantly underway to achieve smart, zero-release of the drugs by capping the surface of the pores. Controlled and intelligent delivery of drugs to the target site through MSNs is possible due to gated release. The gates of the pores open only in response to certain stimuli like pH, temperature, enzyme, redox and so forth. The principle of gated drug release is very effective when toxic side effects of drugs to other organs are to be avoided [204]. Numerous reports in this regard have been published, a few of which we have discussed in the following section.

#### 5.3.1. pH-Responsive Drug Release

pH is the widely explored stimuli to trigger the drug release as the body has a wide range of pH. MCM-48 particles loaded with prednisolone were coated with succinylated ε-polylysine (SPL) to ensure the pH-dependent release of the drug in the colon region. The in vitro release experiments showed a delay in the release of drug which indicated the successful approach of pH-responsive drug delivery. At acidic pH of the stomach, SPL prevents drug release due to its unionized form whereas, at colonic pH, SPL gets converted to its ionized form facilitating drug diffusion out of the MSNs. The developed nanoparticles could be an alternative for the treatment of diseases of the colon (inflammatory bowel disease and cancer) [205]. The promising outcome of pH-responsive MSNs was observed for tumour-targeted therapy and another disease where the pH of the affected area is slightly acidic than the normal tissues. This pH-responsive drug release can be realized by capping the pores of MSN using acid degradable polymers, polyelectrolytes, some pH-sensitive linkers and so forth. pH sensitive polysaccharide, chitosan was coated onto the MSNs to achieve controlled delivery of curcumin for the treatment of cancer. In vitro drug release studies proved the pH-sensitive nature of chitosan by sustained drug release. The release of curcumin improved when moving from pH 7.4 to pH 5.5. Cell uptake studies in U87MG glioblastoma cancer cell-line showed a decrease in the half maximal inhibitory concentration (IC50) values indicating an improved accumulation of curcumin in cancer cells when encapsulated within chitosan loaded MSNs [206]. Similar studies using chitosan as the pH-triggered cap was carried out by Hu et al. for the release of doxorubicin on MCF-7 breast cancer cells. At acidic pH, the amino groups of chitosan become protonated resulting in swelling of the polymer chains. This opens up the pores of MSNs releasing the drugs [207]. Modulation of doxorubicin via pH-responsive stimuli was also achieved using polymers such as poly(acrylic acid) [208] and polydopamine [209]. Both these reports suggested an enhanced uptake by cancer cells and a sustained release of the drugs. Reports with different polyelectrolytes as pH motifs have shown promising outcomes [208,210]. Tannins as pH motifs were proved by the work of Hu and collaborators. Tannins, by the formation of boronate esters were found to modulate the release of the dye rhodamine at acidic pH [211].

**Figure 4:**
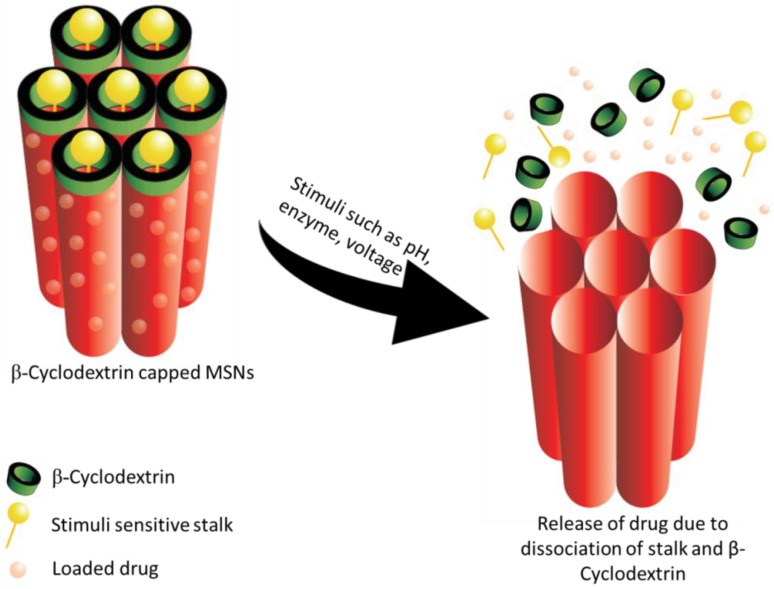
Schematic illustration of the release of drug from β-CD capped MSNs in response to stimuli.

Macromolecular compounds like cyclodextrin (CD) has also been explored for its pH-sensitive property [212]. Tan et al. used a stalk of p-anisidine, loaded the cargo and finally capped the pores with β-CD. The pH sensitivity of the resulting MSNs was evaluated over a pH range of 7.4 to 5.5. A pH-dependent release was observed with a greater release at pH 5.5. When the pH fell below the pKa of the stalk, the interaction between p-anisidine and β-CD reduced thus resulting in the release of the drug. An optimum density of the stalk was the key to control the pH-triggered release. In addition, the effect of both α and β-CD capping on the release of drug was studied. It was observed that the percentage release with α-CD was comparatively lesser that β-CD. This could be due to the different formation constants between CD and p-anisidine stalks [213]. Figure 4 illustrates the release of drug from β-CD capped MSNs in response to stimuli.

Kuthati et al. devised a strategy to ensure the efficient delivery of antimicrobial agent by modifying the MSN framework via pH sensitive MSNs. Institute of Bioengineering and Nanotechnology-4 (IBN-4) nanoparticles, a type of MSNs was used as a carrier. This was immobilized with silver-indole-3-acetic acid hydrazide (IAAH-Ag) via a pH sensitive hydrazine bond. When exposed to the acidic environment of the site of bacterial infection (pH 5.0), the silver ions are preferentially released up to 12 h to ensure controlled release of the model drug by the cleavage of IAAH-Ag coordination bond. The antibacterial efficacy was investigated on two drug resistant strains of gram negative and gram positive bacteria namely *Escherichia coli* and *Staphylococcus aureus* respectively. In addition to this, the effect of IBN-4-IAAH-Ag NPs on inhibiting the formation of biofilms by four strains of bacteria *viz*, *Escherichia coli*, *Bacillus subtilis*, *Staphylococcus aureus* and *Staphylococcus epidermidis* revealed promising results. The in vitro results were substantiated by in vivo experiments on *Escherichia coli* infected C57BL6 mice. A remarkable reduction in *Escherichia coli* was observed in the formulation treated group of mice. Hence the developed system could be a plausible alternative to the antibiotics used currently [214].

#### 5.3.2. Redox Responsive Drug Release

Redox triggering is another widely used strategy to control the release of cargo from carriers. This technique is being used to deliver anticancer drugs making use of endogenously present reducing agents. Redox cleavable disulfide bonds are often utilized for this kind of drug release. Wang et al. synthesized a disulfide-linked polyethylene glycol (PEG) tethered to MSN for redox responsive drug release. The efficiency of the synthesized MSNs was evaluated by in vitro release studies using Rhodamine B (RhB) as the model drug. Glutathione (GSH) equivalent to intracellular concentration was added to the release media. It was observed that in the absence of GSH, the release of RhB was negligible signifying the efficiency of the cap in blocking the drug release. In addition, the PEG surface modification conferred on the nanoparticles a significant biocompatibility [215]. Dual responsive stalks with β-CD caps were synthesized to control the release of multiple drugs. HMSNs with acetal and ferrocene carboxylic acid units were obtained by selective functionalization. The redox responsiveness of the carrier was confirmed by in vitro experiments using hydrogen peroxide as stimuli. On increasing the concentration of hydrogen peroxide, an increase in the release rate of the drug was observed due to the oxidation of the ferrocenyl moiety. The mechanism behind the release is the strong electrostatic repulsion between β-CD and the oxidized hydrophilic ferrocenyl moiety which results in the dissociation of β-CD caps thus releasing the drug from the pores. The same carrier was also made pH-responsive by introducing an acetal linker to develop a synergistic effect [216].

#### 5.3.3. Temperature Responsive Drug Release

Thermoresponsive MSNs have also been widely been studied as a possible means of controlling drug release. In this context, PEO-b-poly (*N*-isopropylacrylamide) based copolymeric micelles as structure directing agents for the synthesis of functionalized MSNs was developed by Bathfield et al. Ibuprofen as a model drug was loaded into the mesopores using one-pot strategy wherein the drug was incorporated directly into the hybrid material. Ultimately, the structure directing agent in this formulation was the drug-loaded polymer micelles. The drug release profile at 20 and 45 °C revealed a temperature sensitive pattern with a higher drug release at 45 °C than that at 20 °C [217].

#### 5.3.4. Chemical and Enzyme Responsive Drug Release

Several chemicals and enzymes present inherently in the body or produced during diseased conditions have also been explored for the possibility of triggering drug release from the MSNs. Glucose as a chemical has been studied by scientists as a possible trigger to release drugs and has been a boon for diabetes management [218]. In one of the studies, MSNs functionalized with a signal reporter, alizarin complexone (ALC) was developed. Gluconated insulin was then introduced within the pores by benzene-1,4-diboronic acid (BA) mediated esterification reaction. This behaved both as a hypoglycaemic agent as well as pore blocker. In addition, rosiglitazone maleate was also introduced into the pores to form multifunctional MSN. In the presence of glucose, competitive binding between ALC and BA occurs which leads to opening of the pores and release of the drug [174]. Similar work for controlling the drug release using two stimuli that is, glucose and pH was carried out by Tan et al. They fabricated glucosamine-poly(acrylic acid) conjugated MSNs. The pores were capped by the crosslinking of the poly(acrylic acid) chains by the formation of boronate esters. The drug release was governed by pH or the presence of glucose. At mild acidic conditions of pH 6.0 and the presence of 10 mM glucose, the drug release was found to be higher by around 65%. These results suggested that at pH 6.0, combined stimuli showed a sufficiently enhanced release of the drug [219]. Another novel chemical sensitive MSN was with that using thrombin. The MSNs were loaded with an anticoagulant drug (acenocoumarol) and the pores were capped with peptide LVPRGSGGLVPRGSGGLVPRGSK-pentanoic acid (P) which is a substrate for proteolytic α-human thrombin. The results demonstrated that the release of drug was highly specific to the presence of thrombin. Thrombin results in the hydrolysis of the capping peptide releasing the drug [220].

Tailoring the drug release in response to the presence of certain enzymes is another new avenue for modifying the MSN as a carrier. NAD(P)H: quinone oxidoreductase 1 (NQO1) enzyme as stimuli for the release of doxorubicin was demonstrated by Gayam et al. They synthesized MSNs surface functionalized successively with alkyne followed by drug loading. To avoid premature drug release, the mesopores were blocked with rotaxane followed by tethering it to benzoquinone. In the presence of NQO1 enzyme which is upregulated in several tumours, the benzoquinone gets reduced leading to the opening of the pores and the release of doxorubicin. In vivo experiments in nude mice bearing tumour (lung) showed promising results with a significant reduction in tumour volume in the mice treated with MSN-NQO1 compared to those treated with saline and free doxorubicin [221]. Matrix metalloproteinase (MMP-2) triggered drug release for the treatment of liver cancer is another possible line of therapy. Many tumours have shown to overexpress MMP-2, the advantage of which could be explored to target drugs to tumour sites. A two-component that is, a cell penetrating peptide polyarginine and PVGLIG which is a substrate for MMP-2 cleaving based polypeptide was linked with phenylboronic acid-human serum albumin (PBA-HSA) onto the MSNs to render it target specific. Doxorubicin (DOX) was used as the model drug. Human serum albumin was used to cap the pores and phenylboronic acid behaved as the targeting moiety specific to sialic acid overexpressed in liver tumours. The enzyme responsive behaviour of the carrier was studied in vitro where around 73% of the drug released in the presence of MMP-2 as compared to only 15% of release in the absence of MMP-2. In vivo studies in HepG2 cells injected nude mice were performed which revealed encouraging results with DOX-loaded MSN-HSA-PBA showing significant tumour growth inhibition compared to non-functionalized MSN loaded with DOX [222]. An experiment along similar line was performed by Radhakrishnan et al. who explored the use of protamine, a peptide drug as a capping agent to prevent the premature release of diclofenac from MSNs. The protamine cap was found to get hydrolysed in the presence of trypsin enzyme which cleaves the l-arginine residues in protamine thereby releasing the drug. This concept was evaluated by studies on COLO 205 cells. The results showed that about 87% of the drug released within 120 min in COLO 205 cells as compared to 13% in healthy cells. This shows the selective drug release in colon cancer cells as compared to normal cells [223]. Radhakrishnan et al. also demonstrated the use of chondroitin sulphate (CHD) as a gate to the pores of MSNs which would trigger the release of drug in the presence of hyaluronidase enzyme. In addition, CHD also acts as a ligand which gets specifically uptaken by cells overexpressing CD44 receptors. CHD behaves as a cap which prevents the outward diffusion of the drug in the absence of hyaluronidase enzyme [191].

In one of the studies, controlled release of drug was achieved by surface functionalization of MSNs with ferrocenyl moiety β-cyclodextrin complex (Fc-β-CD) to prevent premature release of the drug. The experiments suggested that on stimulation by heme protein (horse-radish peroxidase and hydrogen peroxide) and the production of hydrogen peroxide via the oxidation of glucose by glucose oxidase or +1.5 V stimuli, the ferrocenyl moiety gets dissociated resulting in the opening of the nanovalves and thus releasing the drug. In vitro studies conducted to assess the release property of the carrier revealed that on stimulation with heme protein, a greater amount of drug was released as compared to that with glucose stimulation and without stimuli [224].

#### 5.3.5. External Stimuli for Drug Release

**Figure 5:**
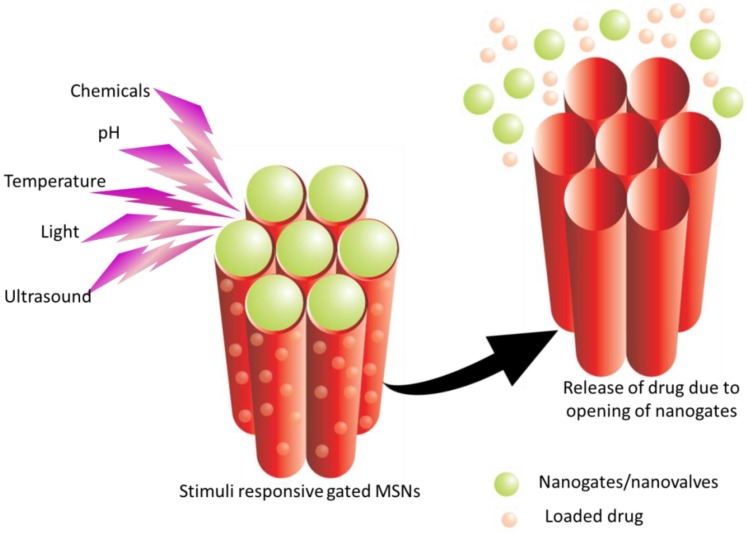
Schematic illustration of the release of drug from gated MSNs in response to stimuli.

Various other stimuli have also been investigated in modulating the drug delivery from MSNs such as light [225,226,227], magnetic [227,228], ultrasound [229,230,231], electroresponsive [224,232] systems. Figure 5 depicts the release of drug from gated MSNs in response to stimuli.

Use of light as an activating mechanism of drug delivery has garnered attention due to its advantages of spatial and temporal control of release of drugs. Febvay et al. [225] combined the advantages of light triggering with a high loading of MSNs in their study to enable the delivery of a model molecule, a fluorescent dye–Alexa546–which is impermeable to the cytosolic compartment. The MSNs were prepared using secondary surfactant pluronic F127 and further tagged with biotin or streptavidin. Following endocytosis, the LN-229 cells (human glioma, ATCC) were exposed to green excitation light for 3 to 120 s which resulted in the dye being released into the cytosol. This could be attributed to the cell membrane damage induced by the light irradiation. Liu et al. [226] explored the potential of deeper tissue penetration potential of near infrared (NIR) radiations to control the release of the drugs. They developed MSNs with gold nanorods forming the inner core and phase changing molecule, 1-tetradecanol as gatekeepers. DOX was chosen as the model drug molecule. These nanoparticles were further functionalized with folate moieties to target KB cells. The system was able to release DOX due to heating induced by near IR radiation generated by a gold nanorod core. The successful release of the drug on IR radiation was ascertained by first subjecting it to external heating which did not show any release at 37 °C. On NIR irradiation of 802 nm for 10 min, the temperature increased to 45 °C which resulted in the release of DOX which may be attributed to the change in the fluid state of tetradecanol molecules above T_m_. In vitro studies on KB cells showed preferential uptake by the cell owing to the surface functionalization. The combined effect of phototherapy (NIR irradiation), chemotherapy (DOX) and targeted therapy resulted in remarkable killing of cancer cells. Hence the developed nanoparticulate system could be efficiently used for multimodal therapy. Li et al. [227] worked on similar lines where they explored the potential of MSNs as multimodal carriers using MRI monitored magnetic targeting and NIR mediated phototherapy. In their work, they developed iron oxide (Fe_3_O_4_) nanoparticles coated with trisoctahedral gold (Au) shell which were loaded with DOX. These were further coated with silica shell capped with oligonucleotides. Au shell here acted as NIR responsive material whereas Fe_3_O_4_ facilitated magnetically triggered release of drug. The controlled release property of the carrier was confirmed via in vitro studies on HeLa cell lines which showed DOX release upon NIR irradiation at 808 nm and magnetic attraction for a brief period of 2 h. These results were further supported by in vivo studies on nude mice. HeLa cells were transplanted to the nude mice for the development of tumour after which the nanoparticles were injected. On exposure to magnetic attraction for 30 min and laser exposure at 3 W/cm^2^ for 30 min, a complete disappearance of tumour was observed after 14 days. These findings suggest the plausible use of these carriers to enable efficient combination therapy options for tumours.

Another novel strategy to induce visible light irradiated delivery of drugs using MSNs as drug carriers was put forth by Kuthati et al. They developed a silver nanoparticle (SNPs) decorated copper impregnated MSNs (Cu-MSNs) to aid in the photodynamic inactivation of antibiotic resistant *Escherichia coli.* Curcumin (Cur), a phototherapeutic agent was loaded into the MSNs. They explored the plasmonic resonance coupling between curcumin and silver nanoparticles to enhance the transfer of silver nanoparticle to photosensitizer to effectively kill gram negative *Escherichia coli*. They hypothesized that curcumin would produce large amounts of ROS under light irradiation which will enhance the release of silver ions. SNP+Cur behaves as a positively charged nanocomposite which improves the binding ability of Cur to the bacterial membrane. The antibacterial efficacy against *Escherichia coli* was studied by constant illumination using LED array at 470 nm at a fluence of 72 J/cm^2^. The study yielded promising results revealing that Cur-Cu-MSN-SNP efficiently killed bacteria in light conditions with very mild toxicity in dark conditions. The successful eradication of bacterial cells may be attributed to three factors, namely, improved solubility and local concentration of Cur loaded into MSNs, improved binding of positively charged nanoparticle to bacterial membrane, enhanced ROS production due to Cu-MSN-SNPs. Similar strategy can be used for the development of wide variety of photobactericidal systems [233].

The release of drugs from MSNs can also be triggered via magnetic attraction. Baeza et al. [228] reported the synthesis of a hybrid polymer which responded to both thermal and magnetic stimuli. They incorporated superparamagnetic iron-oxide nanocrystals into the mesopores which was capable of providing a sufficient heating capacity for hypothermia cancer therapy. To enable thermoresponsive release of the drug molecule, poly(*N*-isopropylacrylamide) was tethered to the surface of MSNs. Fluorescein and soybean trypsin inhibitor type II was loaded into the pores of the MSNs to model the release property. In response to an alternating magnetic field of 24 kA/m and 100 kHz inside a thermostatic chamber maintained at 20 °C, a higher release of fluorescein was observed which may be due to the heat energy and enlargement of the pores leading to greater release of the drugs.

Among the non-invasive routes to enhance the spatio-temporal delivery of drugs, ultrasound is slowly gaining popularity owing to its advantages of lower cost, absence of ionizing radiations and ease of tissue penetration regulation by tuning the frequency of the cycles and exposure time. They are capable of inducing thermal/mechanical effects which can trigger the release of the drugs. Several efforts are underway to utilize this technology for the therapy of diseases especially cancer. Paris et al. formulated an ultrasound responsive MSNs capped with the copolymer, poly(2-(2-methoxyethoxy) ethyl methacrylate-co-2-tetrahydropyranyl methacrylate), with two functional ends and a monomer ratio 90:10 (MEO_2_MA:THPMA). They utilized fluorescein as the model molecule to monitor the developed system. Ultrasound irradiation resulted in the cleavage of the hydrophobic tetrahydropyranyl moiety of the cap leading to a change in its conformation allowing the drug to be released. They studied this concept further by surface functionalization of the MSNs with biotin and RGD peptide to enable selective uptake by tumour cells (HeLa cell lines). DOX was loaded as the cargo within the mesopores. They successfully proved the efficiency of the developed nanoparticulate system in enhancing the efficiency of the DOX in killing cancer cells [229,231]. Studies on similar line was reported by Kim et al. [230] using ibuprofen as a model drug which were loaded into MSNs and covered with poly(dimethylsiloxane) as the implantable body. On exposure to ultrasound of 28 kHz with a power of 1.5 W/cm^2^, an increase in the diffusion of ibuprofen was observed. In addition, negligible damage was observed to the polymer material on subjecting it to ultrasound frequency. These results suggest the possible use of this stimulus in enabling controlled release of drug to the diseased sites.

Another interesting approach to non-invasive drug therapy could be the use of a mild electric field that aids in controlling the release of the cargo by activating certain mechanisms, *viz*, electrochemical reduction-oxidation and movement of a charged molecule. Xiao et al. [224] developed a novel controlled release MSNs sensitive to enzyme or voltage. These were functionalized with ferrocene and further loaded with rhodamine and capped with β-cyclodextrin (β-CD). Voltage ranging from 0.5 V to 1.5 V was applied to trigger the release of the cargo from the pores. Both the enzyme and voltage triggered release was based on the presence of ferrocenyl (Fc) and β-CD valve. The release is based on the conversion of Fc to Fc^+^ which dissociates from the surface of the pores under a standard potential of +0.32 V. They observed that at a higher voltage, the release rate was higher and vice versa. Wang et al. [232] also utilized the potential of ferrocene functionalized β-CD as nanovalves to modulate the drug release from MSNs. They loaded two drugs that is, gemcitabine and doxorubicin and evaluated the potential of ferrocene in controlling the drug release. On applying a voltage of +1.5 V, a controlled release of 23% of gemcitabine was observed over 15 min. Thereafter −1.5 V was applied, which ceased the release of the drug. Hence, this approach can be effectively used for controlling the drug release from MSNs and thus avoiding side effects.

### 5.4. MSNs for Improvement of Solubility of Drugs

MSNs owing to their modifiable surface chemistry can act as carriers for poorly soluble drugs and tackle their solubility issues [234]. Bukara et al. [235] proved this potential of MSNs by loading the poorly soluble drug fenofibrate and assessing them in healthy human volunteers. The volunteers were monitored for a period of 96 h post dosing and their plasma samples were collected and assessed for the pharmacologically active metabolite fenofibric acid. A significant increase in C_max_ with a point estimate of 177% and a reduction in t_max_ were observed for fenofibrate formulation following single oral administration. No serious adverse events were reported and none of the volunteers discontinued the study. This demonstrates that the MSNs could also be used as a possible alternative to other carriers to improve the solubility and bioavailability of drugs. Enhanced oral bioavailability of telmisartan (TEL) was achieved by loading it into MSNs. Based on the results obtained by the study on beagle dogs, Zhang et al. set forth a basis to use MSNs as a drug carrier for poorly soluble drugs. In vitro cellular uptake studies showed that TEL-MSN showed an enhanced uptake in Caco-2 cells resulting in accumulation in the cell membrane as compared to TEL-mesoporous silica microparticles. The uptake mechanism of these MSNs occurred in three major steps: first, binding of MSNs to intestinal cell membrane followed by nonspecific cellular uptake and merging with endosomes and finally, release from endosomes and enters the cytoplasm. In vivo absorption studies of TEL-MSN in beagle dogs revealed a 1.29 times increase in AUC_0→72h_ as compared to that of marketed tablet and MSN [236]. Thomas et al. synthesized MSNs loaded with BSC-II antiepileptic drugs, carbamazepine (CBZ), oxcarbazepine (OXC) and rufinamide (RFN). The dissolution profile of the drugs in phosphate buffer, showed faster release for CBZ and OXC whereas RFN showed a slower release of drugs after an initial burst release. The profile resembled a first order release mechanism related to the drug diffusion process. This could be widely exploited for the improvement in drug absorption and bioavailability of poorly soluble drugs by further proving this concept via in vivo studies [237].

### 5.5. MSNs in Biomedical Imaging and Theranostic Purpose

The versatile features of MSNs such as ability to incorporate wide variety and large number of compounds within them, their stability and controllable size, makes it an ideal platform for biomedical imaging and theranostic applications. Many of the fluorophores face certain drawbacks such as poor solubility and stability especially those for near infrared (NIR) imaging. These compounds can be incorporated within the MSNs to improve their photophysical and photochemical properties. Various literatures have suggested the successful utilization of this carrier for imaging and theranostic purposes.

MSNs can be widely used for optical imaging, magnetic resonance imaging (MRI), positron emission tomography (PET). Optical imaging is a technique wherein the specific probes are excited by incident light usually in the visible or near infrared regions, thus emitting light at a lower energy. MRI is a powerful in vivo imaging technique which gives a three dimensional anatomical picture of the region of interest with a high resolution.

Most often, the dyes are incorporated within the mesopores which gives sufficient stability and protection from the external environment. Sreejith et al. developed a novel hybrid material constituting squaraine loaded MSNs which were further coated with thin sheets of graphene oxide. Squaraine dyes possess significant photophysical properties in the NIR region. In order to ascertain their bioimaging ability, in vitro studies were carried out on HeLa cell lines which showed positive results. The developed hybrid formulation was successful in protecting the dye as well as preventing its leakage from the system showing a potential platform for bioimaging [238]. Moreover, these MSNs can also be tagged with surface active moieties which can be preferentially directed to abnormal tissues for diagnostic and therapeutic purposes. Nakamura et al. reported the synthesis of multimodal MSNs possessing features of imaging as well as drug delivery. The carrier was loaded with ^19^F, a MRI contrast reagent. These nanoparticles were further labeled with fluorescent dyes and tethered with folic acid to ensure adequate uptake by tumour cells. The developed nanoparticles demonstrated positive results in vitro exhibiting sufficient cellular uptake by folate expressing cancer cells which was observed via ^19^F MRI and fluorescence microscopy [239]. Jun et al. reported the use of silica nanoparticles embedded with quantum dots (QDs) and constituting a core-shell of CdSe@ZnS for bioimaging purposes (Si@QDs@Si NPs). They observed that the system showed superior fluorescence as compared to single quantum dots when studied in HeLa cells. The same was confirmed via in vivo testing wherein the mice were injected with Si@QDs@Si NPs mixed with HeLa cells. The nanoparticles exhibited enhanced fluorescence and hence can be effectively used for bioimaging which requires minute cell tracking with high sensitivity [240]. Helle et al. explored the potential of cyanine 7-doped silica nanoparticles for lymph node mapping using NIR imaging. They were able to map the lymph nodes for the diagnosis of possible metastatic and draining nodes. In addition, the developed platform were found to be excreted via hepatobiliary route and was found to be safe when tested in mice up to a period of three months showing an efficient hepatobiliary excretion [241].

### 5.6. MSNs for Bone Tissue Engineering and Repair

MSNs have found a special place in the field of tissue engineering. Most of the research in this area revolves around bone tissue differentiation and osteogenesis. The surface silanol groups present on MSNs react with the body fluids to generate carbonated apatite which can further lead to bone generation. In addition to this, the MSNs can be loaded with osteogenic agents to augment the bone tissue engineering process [242,243]. For instance, bone morphogenetic protein-2 (BMP2) derived peptide functionalized dexamethasone loaded MSNs were formulated to evaluate its efficacy for the osteogenic differentiation. The evaluation was carried out using cell line studies to study the endocytosis and uptake of the functionalized MSNs. The ectopic bone formation was studied in vivo, the results of which indicated that the BMP2-pep functionalized MSNs held great promise in bone repair. The addition of dexamethasone synergized the bone differentiation effect [177]. Similar results were reported by Luo et al. for bone forming peptide incorporated MSNs [244]. Incorporation of bioactive glasses into mesoporous silica is another interesting application of MSN in bone repair. Increased bone tissue regeneration was observed with these materials containing SiO_2_-CaO-P_2_O_5_ as composition. Readers are directed to references [245,246] for a detailed review of these materials.

## 6. Biodistribution and Biocompatibility of MSNs

The safety and toxicity of nanoparticles are a cause of major concern owing to their high surface-to-volume ratio compared to its counterparts. The biocompatibility of any carrier is a prerequisite property for any pharmaceutical product to ascertain that these products do not accumulate in the body over a period of time causing untoward effects.

Many of the formulations containing conventional nanocarriers have been approved by US FDA (Table 1). Biomaterials such as lipids and polymers constitute these conventional nanocarriers. Due to its inherent biodegradability and biocompatibility, these nanocarriers have been constantly exploited for further research to enhance its biomedical applicability. Liposomes are one such carrier which comprises of phospholipid bilayer within which both hydrophobic and hydrophilic drug can be encapsulated. Liposomes have proven to be capable of being used for site specific drug targeting in a variety of diseases [247,248]. These carriers have been found to be safe which may be attributed to the biocompatible nature of phospholipids used [249]. Another nanocarrier that shares a similar importance to that of liposomes is the polymeric nanocarrier. In this regard, poly(lactic-co-glycolic acid) (PLGA) is one of the renowned polymer-based carriers. This is a part of an FDA approved device [249,250]. Although these encouraging results have been obtained regarding their safety for human use, there are certain drawbacks such as stability related issues, lack of control over drug release and difficulty in overcoming certain biological barriers associated with these carriers. Nevertheless, these are still the most widely explored carriers due to their non-toxic property.

Inorganic nanocarriers with robust characteristics, MSNs, although has shown positive in vitro results, studies are still being carried out extensively due to the age-old toxicity-related issues of silicon dioxide, especially silicosis. Efforts are underway in identifying the major routes of toxicity of silica in both its crystalline as well as amorphous forms. In this section, we have focused on the current data available on the biodistribution and biocompatibility of MSNs. Control over the size, shape, pore order and surface chemistry is crucial in deciding the fate of MSNs.

### 6.1. Effect of Surface Chemistry, Shape, and Size of MSNs

As per the reports, the major pathway of toxicity associated with silica is due to its surface chemistry (silanol groups) which can interact with the membrane components leading to the lysis of the cells and leaking of the cellular components [251,252]. Mesoporous silica exhibited lower hemolytic effect compared to non-porous silica. This could be attributed to the lower density of silanol groups on the surface of mesoporous structures [94]. Surface properties of MSNs also have a great impact on the biodistribution and biocompatibility of MSNs. Altering the surface features by functionalization with polyethylene glycol (PEG) helps the MSNs to escape from being captured by liver, spleen and lung tissues. This could be attributed to the longer circulation time of PEG-MSNs [253]. Yu et al. studied the impact of pore size, shape and surface features of silica nanoparticles on the cellular toxicity. The cellular toxicity was evaluated on macrophages (RAW 264.7) and cancer epithelial (A549) cells. Post 72 h exposure, they observed that A549 cells were resistant to the nanoparticles even at the concentration of 500 µg/mL. However, at a concentration of 1000 µg/mL, observable toxicity was seen. The IC50 value for the nanoparticles when tested on macrophages was found to be between 50 and 100 µg/mL. The cellular level of association was determined using inductively coupled plasma mass spectrometry (ICP-MS). Interestingly; it was observed that amino modified MSNs showed a higher level of cellular association which is contradictory to literature which report that increase in surface silanol groups are responsible for higher cellular association [251,252]. The plausible explanation for this higher interaction between amino MSNs and cells could be that a particular surface threshold exists beyond which cell interaction is facilitated. The observations from the above study suggest that toxicity depends on the type of cells, concentration of nanoparticles treated and pore size and surface charge of nanoparticles [254].

An interesting experiment to identify the effect of the spatial arrangement of MSN surface amine groups on its interaction pattern with cells was performed by Townson et al. MSNs with the same size, porosity and charge were modified with suitable reagents (trimethoxysilylpropyl modified polyethyleneimine, 2-[methoxy(polyethyleneoxy)propyl]trimethoxysilane, *N*-trimethoxysilylpropyl-*N*,*N*,*N*-trimethyl ammonium chloride) resulting in PEG-PEI and PEG-NMe_3_^+^ MSNs to ensure exposed polyamines and distributed, obstructed amine groups respectively. Both in vitro and in vivo experiments were performed to determine the toxicity effects. The synthesized nanoparticles were subjected to cytotoxicity studies on a wide range of cell lines such as A549 (human lung carcinoma), A431 (human epithelial cancer), Hep3B (human hepatocellular carcinoma) and human hepatocytes. PEG-PEI MSNs were found to bind to all the cells whereas PEG-NMe_3_^+^ MSNs showed limited binding. To confirm these results and to ascertain if the same effects will be observed in a biological system as well, 50 µg was injected into the veins of ex vivo chick embryos which helped in real-time imaging of particles. The results showed a similar trend as that observed in in vitro studies. PEG-PEI MSNs were found to bind endothelial cells and stationary and circulating white blood cells (WBCs) whereas PEG-NMe_3_^+^ MSNs remained in circulation for >6 h. In order to verify the importance of amine groups in binding, the MSNs were subjected to acetylation thereby shielding the amine groups. This reduces the binding affinity of the MSNs. From the study, it was concluded that exposure to charged particles and its effect on the formation of protein corona in vivo should also be considered when designing MSNs for biomedical applications [255].

The biodegradation and toxicity of MSNs also depend on the shape of the MSNs. The effect of shape on in vivo toxicity of MSNs after oral administration was studied by Li et al. for MSNs with different aspect ratios of 1, 1.75 and 5. These MSNs were administered at a dose of 40 mg/kg. Two hours post administration, a significant number of MSNs were observed in the liver and spleen. The number of spherical nanoparticles showed a marked increase in the liver compared to its rod-shaped counterparts. It was observed that MSNs showed a rapid excretion from the body via faeces while some of the unchanged MSNs or their degradation products could be absorbed and later excreted via urine. The in vitro results of degradation showed that spherical nanoparticles showed rapid degradation while long rods have slow degradation rate, especially in intestinal fluid. These results suggest that degradation of MSNs depends on the shape and biological environment. No abnormalities were observed in liver, spleen, lung and heart. However, spherical nanoparticles induced renal tubular necrosis and haemorrhage which may be due to the degradation products. Contrasting results were obtained for MSNs administered via an intravenous route where no major abnormality in kidney was detected [132,256]. However, there is a lack of clear picture of the degradation pathway and pharmacokinetics of MSNs. With this regard, efforts were made by Zhao et al. to study the pharmacokinetics and biodistribution of different shapes of MSNs namely, long rod, short rod and spherical particles following oral administration. The retention of the nanoparticles in the gastrointestinal tract was determined by ex vivo optical imaging method. In vitro cytotoxicity study revealed that all the three nanoparticles were nearly non-toxic in nature which can be attributed to the conversion of nanoparticles to non-toxic silicate ions. To predict the biodistribution of the nanoparticles, Si content in different organs was determined by inductively coupled plasma optical emission spectrometry (ICP-OES). On examining the Si content in different organs, Zhao et al. also arrived at the same conclusion as that by Li [256], that majority of the MSNs accumulate in the liver. The synthesized MSNs did not show any visible histopathological changes when compared with that of the control indicating that the particles did not produce any gastrointestinal toxicity or inflammation [257].

Size of the nanoparticles also has a profound influence on the biodistribution and excretion of MSNs. MSNs with a varying particle size from 80 to 360 nm was prepared and their biodistribution was assessed in ICR mice. An increase in particle size led to an increase in its accumulation in the liver and spleen following intravenous (i.v.) administration. However, no pathological abnormalities were observed at the end of 1 month. Smaller sized MSNs undergo slower degradation as it can escape the degradation by liver and spleen [253]. Different types of MSNs (MCM-41, SBA-15) were injected subcutaneously (s.c.) and intraperitoneally (i.p.) to mice. Intraperitoneal injection resulted in the death of animals which may be attributed to the rapid systemic distribution following i.p. injection as compared to s.c. [258]. Acute and sub-acute toxicity profiling of fluorescent mesoporous silica nanoparticles (FMSNs) were performed in female nude mice. 1 mg/mouse/day was administered via the intravenous route. No observable toxicity was seen in the animals. Long-term toxicity study following intraperitoneal injection of FMSNs at a dose of 1 mg/mouse/day twice per week for 2 months was conducted to assess the long-term effects of MSNs. The histopathological examination of body tissues, haematological parameters displayed no apparent changes compared to control. In addition, the FMSNs also showed an enhanced tumour uptake property resulting in a reduction in tumour volume [259]. Single dose toxicity studies by Tang et al. revealed that the nanoparticles exhibited a size-dependent toxicity [260]. Zhang et al. synthesized DOX-loaded MSNs functionalized with folic acid of varying sizes of 48, 72 and 100 nm and investigated the effect of particle size on its in vivo distribution in MDA-MB-231 tumour-bearing Balb/c mice. The animals were sacrificed at the end of 24, 48 and 72 h post injection and their organs were harvested. The amount of Si content in each of the organs was determined by inductively coupled plasma mass spectrometry (ICP-MS). It was observed that MSNs with size of 48 nm showed the highest accumulation in the tumour tissues. The results suggest that particle size and surface modification alters the biodistribution of MSNs [261].

The data generated from various literatures suggest that careful control of particle size and shape is the determinant factor in ascertaining the biodistribution and toxicity of MSNs. In addition, the safety and toxicity of MSNs also depend on the dosage of the MSNs administered at which no observable biological effects are detected.

Recently, Shen et al. [262] reported that the novel 3D-dendritic MSNs synthesized by them showed a rapid biodegradation in simulated body fluid within 24 h as compared to two weeks for that of plain MSNs reported earlier. In yet another effort to prepare biodegradable MSNs, He et al. [263] synthesized a novel pH responsive mesoporous silica–calcium phosphate (MSN-CAP) hybrid nanoparticles. The MSNs were doped with calcium phosphate during synthesis process which yielded pH responsive MSNs. The in vitro degradation behaviour of MSN-CAP was observed in simulated fluids. The results showed that the complete degradation of the nanoparticles took place in 24 h. Both these novel MSNs could be an alternative prospect for clinical use. However, their in vivo degradation behaviour has to be ascertained.

### 6.2. In Vivo Safety and Toxicity of MSNs

Determining the safety and biocompatibility of MSNs is crucial owing to its variable characteristics. Over the last few years, the number of literature on the study of the safety of MSNs has drastically increased. The toxicity of the carriers depends on its various characteristics and the conclusions derived from the studies were found to vary. Nevertheless, most of the reports showed that the MSNs get preferentially accumulated in the liver and spleen following administration.

Liu and collaborators made an attempt to study the single and repeated dose toxicity of HMSNs following intravenous administration in mice. LD_50_ of HMSNs was found to be higher than 1000 mg/kg. In single dose toxicity studies, mice were injected with HMSNs at a low dose and high dose. At the higher dose of 1280 mg/kg, mice did not survive. In contrast, the groups treated with low dose HMSNs did not show any behavioural changes nor any haematology or pathological changes. To carry out the detailed repeated dose toxicity studies, intravenous administration of HMSNs were given to mice continuously for 14 days and observed for a month. During the 1 month observation period, no mortality was observed. Moreover, no remarkable changes in pathology or blood parameters were observed. In order to assess the fate of the nanoparticles, HMSNs were injected intravenously at a dose of 80 mg/kg. Following administration, the majority of the nanoparticles were found to localize in the liver and spleen. Analysis of the silicon content using ICP-OES revealed that highest amount of silica was present in the spleen and liver which gradually reduced over a period of 4 weeks [264].

In order to assess the fate of MSNs after different administration routes, Fu and collaborators tested MSNs with a particle size of 110 nm in ICR mice. Following administration via hypodermic, intramuscular and intravenous injection as well as oral administration, the in vivo distribution of fluorescent-tagged MSNs was tracked. It was observed that of all the exposure routes, the oral route was found to be well tolerated even when the dose was increased to 5000 mg/kg and intravenous route seemed to have the least threshold. MSNs administered via intravenous route were found to preferentially accumulate in the liver and spleen at the end of 24 h and 7 days whereas those administered by other routes did not show any fluorescence in these organs. It was observed that a portion of the MSNs administered via intramuscular and hypodermic route could cross different biological barriers with a slow absorption rate. The major routes of excretion of MSNs were found to be via urine and faeces with the highest values after oral administration as compared to other routes. No histopathological changes were observed in liver, spleen, kidney and lung at the end of 24 h and 7 days by different exposure routes. Nonetheless, a low degree of inflammation was seen in the mice which were treated with MSNs via the hypodermic and intramuscular route. The results suggested that MSNs were found to be safe and well tolerated when administered by oral and intravenous routes [265]. For an extensive review on the biocompatibility of MSNs and silica NPs, the readers are directed to references [260,266,267].

### 6.3. MSNs v/s Silica Nanoparticles

Different forms of silica *viz*, fumed silica, porous silica and non-porous silica can be used as drug carriers. These carriers have shown encouraging results in preclinical studies. However, to translate these materials to the bedside, a clear understanding of the fate and the inherent toxicity of these carriers in vivo is essential. Silica nanoparticles used for biomedical applications are usually amorphous in nature belonging to either porous or non-porous category. The rapid clearance of amorphous silica from the lung compared to the crystalline forms is responsible for its lower toxicity potential [268]. MSNs are found to dissolve rapidly when it is sufficiently below the saturation levels. As per the reports of Martin [269], silica dissolves in the body fluids which subsequently gets absorbed or excreted as silicic acid in the urine. The silica nanoparticles undergo degradation to silicic acid which is non-toxic via three different processes *viz*, hydration, hydrolysis and ion-exchange. This process of degradation was found to depend on the degradation medium and the concentration of nanoparticles. Various strategies have been explored which can manipulate the degradation kinetics of silica nanoparticles. Some of the approaches are noncovalent doping of organic moieties to accelerate the hydrolytic degradation, covalent binding of organically bridged silsesquioxanes- based NPs, and incorporation of cleavable organically bridged silsesquioxanes into silica NPs to enhance the degradation by the biological trigger. The degradation of MSNs is much more complicated than other silica NPs owing to its varying matrix. This is attributed to the difference in the rate and degree of condensation of silica matrices between the various sol-gel procedures of MSN synthesis. As per the review by Croissant et al., partially condensed MSNs degrade in a few days, well condensed MSNs tend to degrade in weeks and calcined MSNs takes months for its degradation [270].

In order to assess the impact of porous structures on the in vivo immunotoxicity, Lee et al. performed repeated-dose toxicity studies on BALB/c mice. MSNs and colloidal silica NPs were injected intraperitoneally into mice for 4 weeks. At the end of the study period, the animals were sacrificed and organs were harvested to study the effects of the particles. The animals treated with MSNs showed an increase in the relative weight of liver and spleen and an increased response to lymphocyte mitogens, concanavalin A (Con A) or lipopolysaccharide (LPS). In addition, a decrease in the CD4^+^/CD8^−^ and CD4^−^/CD8^+^ phenotypes and an increase in the levels of CD4^+^/CD8^−^ and CD4^+^/CD8^+^ were recorded. Elevated IgG/IgM levels were also observed in the MSN treated animals. The results indicate that MSNs showed a greater extent of damage than colloidal silica NPs [271]. However, a careful study of the toxicity is needed to establish the safety of MSNs.

### 6.4. Biocompatibility of MSNs in Humans

The biocompatibility of silica nanoparticles has long been a topic of controversy as studies conducted by researchers have yielded variable results. Nevertheless, the Food and Drug Administration (FDA) approval of hybrid silica nanoparticles for bioimaging marks an event of utmost importance. These particles were found to be ~7 nm in size within which fluorescent dye, Cy5 was incorporated. These particles were labelled with ^124^I and surface functionalized with peptide cyclo-(Arg-Gly-Asp-Tyr) (cRGDY) to selectively target integrin-expressing tumours. C dots were synthesized in such a way that they had limited reticuloendothelial system (RES) uptake and promote renal excretion. Preliminary experiments on in vivo safety by Choi et al. (Cornell University) revealed that these fluorescent silica nanoparticles were safe and did not show any toxicity in mice. These particles were also found to be an effective bioimaging probe for cancer imaging [21,22]. Burns et al. carried out in vivo biodistribution studies of the developed C dots in nude mice wherein nanoparticles were injected intravenously. The particles were found to show rapid renal clearance within 45 min of injection and majority of these particles accumulated in the liver. To further modify the clearance, the particles were coated with methoxy-terminated poly(ethylene glycol) chains. By careful manipulation of the surface features of C dots, they can be used for wide variety of biomedical applications including imaging and therapy [22]. Based on the encouraging results of pre-clinical studies, these nanoparticles received approval from the FDA as an Investigational New Drug (IND) to conduct the human clinical trial, phase I. The first human clinical trials suggested its safety for human use. A pilot clinical trial was conducted in five metastatic melanoma patients to assess the pharmacokinetic (PK) profile of C dots following a single injection dose. The PK profiles, renal excretion, metabolic profile assessment in patients suggested that the particles were well tolerated, preferentially accumulated in the tumour site and were found to be safe for human use [272,273].

A study wherein the potential of ordered mesoporous silica nanoparticles (OMS) in enhancing the bioavailability of fenofibrate in man was conducted by Bukara and collaborators which can be considered as another breakthrough step acting as a trigger in evoking interest among the researchers for the use of MSNs for biomedical applications. Promising results obtained by them in their preclinical studies [274] prompted them to complement those results with clinical studies. Fenofibrate was loaded into OMS and these were subsequently enclosed within capsules. The study was carried out with 12 volunteers who were administered a single dose of fenofibrate OMS and the marketed formulation of fenofibrate, Lipanthyl^®^. Safety assessment was performed by periodic monitoring of the vital signs, 12-lead electrocardiogram (ECG) and blood biochemical parameters in the subjects. The PK profile revealed an increase in the rate and extent of absorption of fenofibrate when incorporated in OMS as compared to the marketed product. In addition, the formulation was found to be well tolerated in the volunteers ensuring the safety of the developed OMS formulation [235].

## 7. Recent Patents Filed in the Field of MSNs for Biomedical Applications

Ever since its first production, modifications in terms of synthesis aiming to control the particle size and pore volume has led to the filing of several patents on MSNs. Owing to its versatile nature of loading therapeutic agents, both hydrophilic and hydrophobic, patents filed on MSNs mainly include investigating them for biomedical applications, biosensors, imaging and as adsorbents. In the following section, we have laid emphasis on reviewing the recent patents related to the biomedical applications of MSNs.

**Figure 6:**
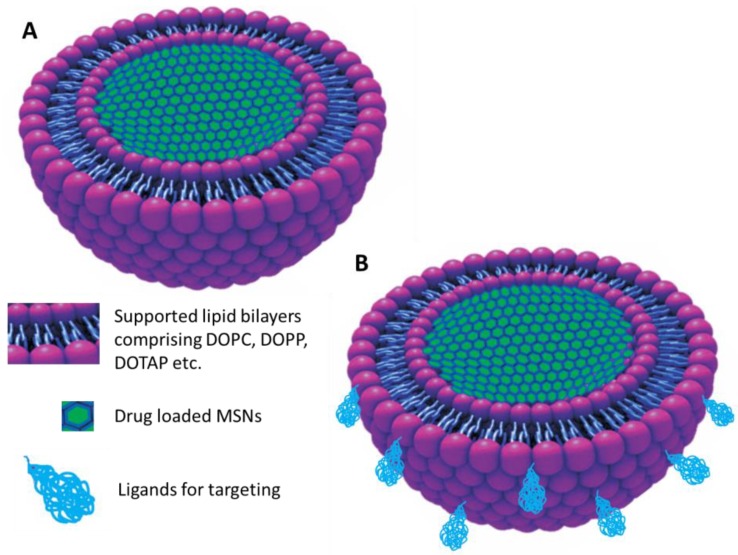
Representation of (**A**) Non-targeted protocell and (**B**) Targeted protocell.

A novel approach of coating the MSNs with lipids coined as ‘protocells’ has received significant attention in the fabrication of drug delivery systems. These combine the advantages of liposomes (low toxicity, long circulation times) with the advantages of MSNs (tunable size, shape and loading capacity) (Figure 6). Numerous studies have shown positive results, a few of which are touched upon here.

A protocell of MSN encapsulated within lipid bilayer was designed by Ashley and collaborators wherein, MSNs were prepared by aerosol-assisted evaporation-induced self-assembly (EISA) procedure. The MSNs were encapsulated within supported lipid bilayers. The lipid bilayer components (cholesterol and phospholipids) were covalently attached to the glycidoxypropylsilane or APTES functionalized MSNs. Levofloxacin was loaded into the pores of the MSNs. The lipid bilayers were composed of either 1,2-dioleoyl-sn-glycero-3-phosphocholine (DOPC), l,2-dioleoyl-sn-glycero-3-phosphoethanolamine (DOPE) or l,2-distearoyl-sn-glycero-3-phosphocholine (DSPC). The protocells were rendered target specific by anchoring peptides (e.g., RGD peptide comprising of Arg-Gly-Asp) onto its surface. The protocells were PEGylated with polyethylene glycol to enhance its circulation time in the body. The pore size of the MSNs ranged from 1 nm to 75 nm. A high drug loading of about 20–55 wt % of the protocell was obtained for individual antibiotics. Fcy targeted protocells were found to show enhanced uptake by THP-1 cells resulting in the effective killing of intracellular organism *F. tularensis*. Only 2 wt % levofloxacin loaded protocell was also found to be cytotoxic as compared to that of free levofloxacin. Biodistribution studies in Balb/c mice showed that Fcy targeted protocells were distributed in various organs of potential *F. tularensis* infection such as lung, liver, spleen and lymph nodes. The results revealed that the biodistribution like many other nanoparticles depends on their size and size distribution. Based on literature reports and approximation of previous work on other antibiotics, the inventors also claimed that oral administration of protocells was far more effective than inhalation therapy for respiratory tularemia. However, these protocells have to be filled in capsules coated with a suitable polymer to prevent its degradation from the gastric environment. The application of protocells could also be extended to incorporate various other antibiotics, macromolecules such as DNA and histone packaged plasmid into the protocells to enhance its penetration into the nucleus of a cell and deposit its contents [275].

Jeffrey and collaborators fabricated MSNs which were functionalized with targeting ligands specific to white blood cells or arterial, venous or capillary vessels. These targeting ligands were either Fc gamma from IgG, human complement C3, ephrin B2 and SP94 peptide. These MSNs were further encapsulated within lipid bi- or multi-layers to form protocells. Polyethyleneglycol-polyethyleneimine (PEG-PEI) was tethered to the surface of MSNs to enhance the colloidal stability of the formulation. The MSNs were around 50 nm in size and positively charged. This helps to bind itself to endothelial cells, serum proteins and white blood cells. To elucidate the binding of these MSNs, they were injected into veins of ex vivo chick embryos. PEI-PEG-MSNs were found to be bound to endothelial cells as well as stationary and circulating white blood cells following injection [276].

Similar use of protocells was extended and reported for multicomponent delivery of drugs DOX, 5-fluorouracil and cisplatin to cancer cells. The protocells constituted MSNs which were surrounded by lipid bilayers also referred to as ‘supported lipid bilayer’ (SLB) and further functionalized with targeting ligand SP94 peptides which are overexpressed in liver cancer. These ligands were conjugated on the surface of amino-modified MSNs by a PEG spacer. The lipids chosen were *N*-[1-(2,3-Dioleoyloxy)propyl]-*N*,*N*,*N*-trimethylammonium methyl-sulfate (DOTAP), 1,2-dioleoyl-sn-glycero-3-phospho-L-serine (DOPS) which functions as a pore sealing agent and thereby restricts the release of the drug. Once internalized by the cells, the SLBs get destabilized by endosome acidification, thus releasing the drug. The protocells could also be tethered with nuclear localization sequence (NLS) to enhance the penetration of the drug into the nucleus of the cells. In vitro cellular uptake of the protocells by Hep3B cells (hepatocellular carcinoma) supported the hypothesis of active targeting by the developed protocells [277].

Toroidal MSNs were synthesized and their potential use as a carrier for the transport of different cargos ranging from small molecules to siRNA, mRNA and plasmids was explored by Brinker and Lin. Both ellipsoidal (eMSN) and toroidal shaped MSNs (tMSNs) were synthesized by varying the reaction procedure and the reactants. ‘Torus’ shaped MSNs refer to MSN with a central pore and two other pores into which macromolecules can be easily loaded. The internal surface area was found to be in the range of 1.1 to 0.5 cc/g with a payload of 50%. These were functionalized with amino groups and modified with PEG to improve the circulation time. Ligands, Fc gamma from IgG, human complement C3, ephrin B2 and SP94 peptide were tethered onto the MSNs for target specificity. DOPC and DOPP (dioctylphenylphosphonate) lipids can be coated onto the MSNs to seal the pores and also improve the biocompatibility. Cellular uptake studies were performed on a variety of cell lines such as human endothelial cells like EAhy 926, ATCC-CRL-2922 and mouse macrophages ATCC-TIB-71 and Raw 264-7. The successful internalization of the MSNs was proved by the in vivo studies. The developed MSNs can be a possible carrier to load large linear molecules due to its unique structure. They can also be loaded with a variety of small molecules ranging from anti-cancer, anti-inflammatory, antiviral and so forth [278].

Protocells for the efficient delivery of chemotherapeutic agents for the treatment of hepatocellular carcinoma was formulated. The protocells contained nanoparticles protected by supported lipid bilayers comprising of DOTAP, DOPG (1,2-dioleoyl-sn-glycero-3-phosphoglycerol) or DOPE as lipids. The nanoparticles were loaded with DOX, cisplatin and 5-fluorouracil as model cargo. The surface of the protocells was tethered to a novel binding peptide c-MET. The pores can also be loaded with small interfering RNA, microRNA. The surface of the nanoparticles was modified with amino groups to sustain the drug release. These were coated with liposomes by electrostatically fusing them to nanoporous silica core. The surface was coated with a fusogenic peptide that promotes the endosomal escape of protocells. They can also be coated with nuclear localization sequence to enhance the uptake by the nucleus. The applicability of these protocells can also be extended for the transdermal delivery of the drugs wherein the supported lipid bilayers contained permeation enhancers to enhance the permeability via the stratum corneum [279].

Nel et al. developed phospholipid bilayer coated MSN loaded with gemcitabine (GEM) for the treatment of human pancreatic ductal adenocarcinoma (PDAC). The MSNs were synthesized and loaded with GEM. The lipid membrane was rehydrated with GEM-MSNs which led to the coating and capping of the pores of the MSNs. In addition to this, paclitaxel was dissolved in the organic solvent along with the lipids thus leading to MSNs with two drugs; one in the pores and other embedded in the lipid bilayer. The loading of GEM into the MSNs was found to be around 20% *w*/*w*. Transforming growth factor β (TGF-β) inhibitor, LY364947 was adsorbed onto MSNs. These TGF-β inhibitors help in enhancing the permeation of GEM laden MSNs to the tumour sites. This proof-of-concept was established in BxPC3 xenograft mouse models (pancreatic tumour model). The delivery system showed enhanced uptake in tumours showing a significant reduction in tumour volume. To prolong the circulation time, the MSNs were coated with PEI/PEG [280].

Silica nanoparticles loaded with antibiotics and their surface coated with a polymer to prevent premature release of the drug were developed by Avni and collaborators. The formulation was designed such that the cargo will be released only in response to stimuli. In this work, the drug within the nanoparticles will be released only if the substance released by the target cell has the property to degrade the gating molecules. Another application of this invention was in the diagnosis of diseases. Signaling molecule loaded silica nanoparticles were gated with nucleic acid molecules. Outside this particle, another molecule which produces a detectable signal was added. In the presence of a nucleic acid which is complementary to the gating molecule, they both hybridize resulting in the opening of the gates of the nanoparticles. In a similar way, the developed nanoparticles were used for various applications by coating the surface of the particles and making it responsive to stimuli [281].

Pore expanded MSNs with a pore diameter ranging from 1 nm to 100 nm for the loading of bioactive material and mainly protein was developed by Cheolhee. The pore expanding agent used was trimethylbenzene. Also, the surface of the MSNs was functionalized with ligands specific to the protein of interest to enhance the binding of the protein either to the inner or outer surface of the carrier. The ligand includes nickel, nickel-nitrilotriacetic acid (NTA), glutathione, dextrin, biotin or streptavidin. The protein of interest in this work was proteasome. Various other proteins such as bovine serum albumin (BSA), IgG proteins, β-galactosidase, horseradish peroxidase were introduced into the pores to study their effect. The surface of the MSNs was further functionalized with the ligand to enhance the intracellular drug delivery using peptides. The intracellular delivery efficiency of the MSNs was studied using fluorescent tagged MSNs. The MSNs were found to show an increased intracellular delivery of the agents as compared to the free proteins. The internalization mechanism of MSN-proteasome complex was studied in HeLa cell lines. It was observed that the complex exhibited energy-dependent caveolae-mediated and clathrin-mediated endocytosis. This drug delivery carrier could be further extended for the delivery of various other proteins and enzymes such as RNase, kinase, phosphatase, antibodies, miRNA or siRNA [282].

Weng et al. worked on improving the efficacy of a natural molecule, 16-hydroxy-cleroda-3,13-dine-15,16-olide (HCD) for the treatment of cancer. Even though HCD has shown great potential in inducing apoptosis, its use is limited by its poor solubility. HCD was incorporated into copper modified silica nanoparticles. To further prolong the release of the drug from the carrier, these nanoparticles were coated with Eudragit^®^S100 (Cu-MSN-HCD-S100). The loading of the drug was found to be around 18% of the weight of the carrier which was supported by the reduction in surface area with each coating. In vitro release profile of the drug showed a sustained release with Cu-MSN-HCD-S100 as compared to the uncoated MSNs. The cytotoxic potential of the developed formulation was observed in rat G6 glioma cell lines. These results were supported by in vivo studies in tumour xenograft C6 rat glioma bearing mouse models which showed a reduction in the tumour volume on oral administration of the formulation. The formulation was found to be safe without any major reported toxic effects [283].

Modified MSNs were fabricated by Lee and collaborators to monitor the redox-responsive drug release within the system. To validate this concept, doxorubicin-loaded MSN labeled with coumarin and tethered to cysteine was developed. The release of the drug was blocked by fluorescein isothiocyanate-β-cyclodextrin (FITC-β-CD) which was covalently bound to cysteine. These carriers are designated as redox-responsive fluorescent resonance energy transfer-based MSN drug delivery system (FRET-MSNs). These FRET systems have a unique feature of energy transfer between two fluorophores which is sensitive to changes in the donor (Coumarin-labeled cysteine) to acceptor (FITC-β-CD) separation distance. The change in the FRET signal was used to monitor the drug release. When the donor and acceptor are in proximity to MSN surface, a green emission peak at 520 nm was observed (FRET ON). In the presence of glutathione (GSH) which are overexpressed in cancer cells, the disulfide bonds get cleaved resulting in the opening of FITC-β-CD valve and release of drug which shows an increased blue fluorescence at 450 nm corresponding to coumarin (FRET OFF). The pore diameter of these carriers was found to be 2.3 nm with a particle size of around 100 nm. This theory was studied using HeLa cells treated with thioacetic acid (GSH synthesis scavenger) and *N*-ethylmaleimide (GSH scavenger). In the presence of thioacetic acid, a decrease in cell viability, as well as gradual decrease in FRET signal, was observed. The opposite was true in case of *N*-ethylmaleimide. Similarly, the same carrier can be used to monitor the release of a wide variety of drugs by suitably modifying the carrier system [284].

A comparatively new avenue of research for the use of MSNs is in the delivery of antibiotic drugs for the treatment of post-operative osteomyelitis and arthroplasty. Polyacrylate based bone cement materials for effective delivery of antibiotics was designed by Shou-Cang and collaborators. A sustained release of 70% of the active principle over a period of 80 days was observed when compared to only 5% release from the currently marketed antibiotic bone cement formulation, Smart-Set GHV.A co-delivery of antibiotics (gentamicin, vancomycin) and anti-inflammatory (indomethacin, ibuprofen) drug was achieved in the current invention. To formulate MSN based bone cement, the drug was loaded into the MSNs and polyacrylate was added to form a mixture to which monomer, methyl methacrylate was added and polymerized to form the bone cement. It was observed that as the content of MSN was increased from 6 wt % to above; an enhanced drug release was observed which was otherwise restricted to <7% as the majority of the drug would be embedded in the bone cement matrix. In the case of MSN, the drug could be released from the matrix via diffusion from the pores. The developed formulation also exhibited low cytotoxicity to mouse fibroblast cells ensuring the safety of the formulation. The compression strength and bending modulus of the bone cement were similar to that of the commercial product. Hence, the current invention can be used as an alternative to treat osteomyelitis, augmentation of the bone crew and bone-implant interface during joint replacement surgery, as bone filler and bone graft substitute [285].

Liu and Lay [286] reported the formulation of stimuli-responsive hollow silica vesicles coated with interpolymer complex for the delivery of bioactive agents. These carriers contained interpolymer complex where the first polymer PEG was immobilized on the surface and the second polymer, poly(methyl methacrylate) (PMMA) was complexed to the first one via hydrogen bond. The principle behind the release of active agent from the pore was related to a pH of the system. At around pH 5, the second polymer will remain complexed to the first one whereas, at pH 7 and above, the interpolymer complex dissociates releasing the drug. The dissociation of the PEG-PMMA complex was due to the deprotonation of PMMA leading to breaking of hydrogen bonds between methyl methacrylate (MMA) and ethylene glycol (EG). This leads to swelling of the complex and dissolving of anionic PMMA. The hollow silica particles were prepared using polystyrene as template and surface modified with amino groups. Calcein blue was loaded as the model cargo to study the behaviour of the delivery system. The ratio of methyl methacrylate: ethylene glycol was in the ratio of 1:3.4. PMMA of varying molecular weights was tried and 6.5 kDa formed a good complex with PEG as it could easily intercalate within the gaps of PEG chains thus providing flexible, smooth PEG-PMMA complex. The developed formulation was evaluated for the proof of concept by in vitro studies. These can be used for delivery of drugs susceptible to gastric pH and can be given via oral route by suitably formulating with additives.

Zink et al. formulated MSNs with its surface modified with mPEG and further coated with a polymer such as a polyethyleneimine (PEI) for the delivery of siRNA and plasmid DNA. Along with this, phosphonate modified MSNs were also synthesized and loaded with drugs. Also, doxorubicin, paclitaxel was loaded into the MSNs. The polymer chain length can effectively control the toxicity of the synthesized MSNs still maintaining the necessary function. To evaluate the toxicity of PEI as a polymer, MSNs were coated with different molecular weights of PEI polymer like 0.6, 1.2, 1.8, 10 and 25 KD. The cytotoxicity potential of the developed formulation was determined in HEPA-1 cells. The results revealed the absence of any toxicity in particles coated with 0.6, 1.2 and 1.8 KD polymers whereas 10 KD polymer showed toxicity at 50 µg/mL whereas 25 KD polymer showed a decline in MTS (3-(4,5-dimethylthiazol-2-yl)-5-(3-carboxymethoxyphenyl)-2-(4-sulfophenyl)-2H-tetrazolium) activity at more than 12.5 µg/mL. In addition to this, paclitaxel was also loaded into the pores to determine the activity of the carrier. The cellular uptake of the paclitaxel-loaded MSNs was determined in PANC-1 and BxPC3 cells (human pancreatic ductal carcinoma). The MSN-PEI-1.2 KD particles exhibited significant cytotoxicity and cellular uptake of paclitaxel whereas MSN-PEI-25 KD showed slight particle related toxicity at a concentration of 25 µg/mL as compared to that of paclitaxel suspension in aqueous media [287].

Liong and collaborators developed MSNs to carry water-insoluble drugs like camptothecin (CPT) and paclitaxel (PCL) for the treatment of pancreatic carcinoma. To render the nanoparticles magnetic in nature for MR imaging, iron oxide nanocrystals were incorporated into the MSNs. They further loaded hydrophobic chemotherapeutic agents into the pores of MSNs. They modified the synthesis of iron oxide nanocrystals by thermal decomposition of iron-oleate complexes which later were merged with cetyltrimethylammonium bromide (CTAB) by the interaction between the hydrophobic tail of CTAB and hydrophobic oleate ligand. The mesoporous silica was formed around the iron oxide nanocrystals at a temperature of 65–80 °C with vigorous stirring to obtain nanoparticles in the range of 100–200 nm. They also successfully utilized the same method with other inorganic nanoparticles like gold and silver in place of iron. The complete removal of surfactant template was brought about by ion exchange method using ammonium nitrate. To avoid agglomeration of the particles, the surface of MSNs was modified with phosphonate groups (that is, trihydroxy silyl propyl methylphosphonate). On loading the drugs into the pores of MSNs, it was observed that only 30 nmol of the drug was loaded onto 1 mg of nanoparticles. To enhance the cellular uptake by cancer cells, the MSNs were functionalized with folic acid moiety. This study was confirmed by cellular uptake studies in pancreatic cell lines (PANC-1, Capan-1 and AsPC-1), colon cancer cell line (SW480) and stomach cancer cell line (MKN-45). Fluorescent MSN clearly indicated the cytotoxic potential of CPT. They also studied the mechanism of cellular uptake in human pancreatic cell line PANC-1 and hepatoma cell line Hepa-1 cells. The results suggested that the uptake of FMSN takes place via temperature and energy dependent manner. This was confirmed by treating the cells with metabolic inhibitors such as sodium azide/sucrose/bafilomycin A, nocodazole/brefeldin A which inhibited the cellular uptake of FMSNs [288].

Sulfasalazine loaded charged MSNs were fabricated by Lee et al. for the effective therapy against diseases of the lower gastrointestinal tract (inflammatory bowel disease, ulcerative colitis, Crohn’s disease). The surface of MSNs was functionalized with *N*-trimethoxysilylpropyl-*N*,*N*,*N*-trimethylammonium chloride via co-condensation method at varying concentration of 2%, 5%, 8% and 12% *v/v* designated as MSN-TA1, MSN-TA2, MSN-TA3 and MSN-TA4 respectively. Sulfasalazine and a dye named orange II were loaded into the pores of the MSNs. The loading percentage was found to be about 1.7 to 4% in water and DMSO as solvent respectively. The concentration of the dye played a significant role in the adsorption capacity. Higher the concentration of orange II, greater was the adsorption of the dye which suggested diffusion dependent adsorption. However, adsorption was found to be greater with the increased density of TA groups on the surface of MSNs. Similar results were observed with that of sulfasalazine as well. However, the loading efficiency was found to be lesser due to the hydrophobic nature of the drug. pH range of 2–5 was found to be optimum for the loading of the drug as at this pH strong electrostatic attraction was found to be present. The in vitro release profile revealed that MSN-TA4 showed a comparatively slower release of the drug compared to the rest of the modifications and unmodified MSN. Their work indicates that the release and adsorption of the drug onto MSNs could be tailored by tethering TA onto the surface of MSNs [289].

Lin et al. utilized room temperature ionic liquids (RTIL) as a template for the synthesis of MSNs. The organic cation used in this work includes alkylammonium and alkylphosphonium cations and heterocyclic cations like *N*-alkyl pyridinium and *N*,*N*’-dialkyl imidazolium. These organic cations were treated with suitable anions, such as tetrafluoroborate, hexafluorophosphate, halides such as fluoride, chloride, bromide and iodide to form RTIL. The pores of the MSNs were loaded with antimicrobial agents. These agents could also form part of the cationic group of the RTIL. They suggested that to obtain a delayed release of the drug either the antimicrobial ammonium species can be used as the template of MSN or the pores can be reloaded with the antimicrobial quaternary ammonium salts. To control the release of the drug from the template, the surface of the MSNs can be further coated with a polymer like poly (lactic acid) or any bioadhesive polymer to render the MSNs bioadhesive which can further prolong the drug release. In the present invention, MSNs with different shapes such as spheres, ellipsoids, rods and tubes were synthesized using different tetraalkoxysilanes namely 1-tetradecyl-3-methylimidazolium bromide (C_14_MIMBr), 1-hexadecyl-3-methylimidazolium bromide (C_16_MIMBr), 1-octadecyl-3-methylimidazolium bromide (C_18_MIMBr), 1-tetradecyloxymethyl-3-methylimidazolium chloride (C_14_OCMIMCl) and cetyl pyridinium bromide (CPBr) respectively. The pores of the MSNs were capped with certain amino acids to alter the drug release. The antibacterial activity of the developed MSNs was determined by disk diffusion assays, minimal inhibitory concentration (MIC), and minimal bactericidal concentration (MBC) against *Escherichia coli* K12. They were also claimed to be effective against fungi. The inventors also developed cetylpyridinium chloride (CPC) containing MSN formulation for the treatment of an oral volatile sulfur compounds (VSC)-prone condition leading to oral malodor problems. The pore surface was blocked with zinc-binding amino acids such as glutamic acid, histidine and aspartic acid groups. In neutral or weakly basic conditions, CPC molecules slowly diffuse out of the pores and suppress the anaerobic protein digestion activities of gram negative bacteria thus preventing VSC formation. The MSN formulation can be administered via oral, topical or parenteral routes depending on the final use. The MSNs can be further formulated using suitable diluents or carriers to convert it into tablets or topical ointments, gels [290].

## 8. Conclusions

In this review, we have touched upon some exciting research utilizing mesoporous silica nanocarriers as drug delivery systems. Their unique properties of tunable pore size, pore volume, high loading capacity makes them widely exploited nanocarriers. Varying the molar composition of the reactants, type of reactants and the reaction conditions, MSNs with different particle size, shape and pore volume can be obtained. Tailoring the surface properties and pore size of MSNs helps enhance the loading and modify the drug release profile. The major research on MSNs is focused on the use of these in the treatment of cancer wherein variety of ligands can be anchored onto the surface of MSNs due to the ease of functionalization. Moreover, these smart systems can be used to deliver drug at the site of interest by various external and internal stimuli such as pH, temperature, light, chemicals, enzymes, ultrasound and so forth. Review of the patents filed shows that majority of the research focuses on exploring the possible use of protocells (MSNs coated with supported lipid bilayers) for drug and macromolecule delivery. With this kind of systems, it is possible to protect the cargo from the external environment and also achieve ‘zero’ premature release. However, the pharmacokinetics and biodistribution of these carriers vary depending on its characteristics and the route of administration. Implications associated with long-term use of MSNs remain unanswered. This lacuna holds back the technology platform from stepping to the next level of clinical use.

## 9. Current and Future Perspectives

Although FDA has approved only a few nanomedicines for treatment and use in the clinics, these novel systems have been successful in laying a huge impact in the field of disease therapy and have the potential to change the conventional treatment or diagnosis. Ever since the first identification of the potential application of MSNs as carriers for drug delivery, exhaustive research is being carried out to prove the importance of this technology in the therapy of multiple diseases. Majority of the work focuses on the use of these carriers for site-specific delivery of chemotherapeutic agents. Nonetheless, regulatory and technical obstacles limit the safe and efficient translation and regulatory approval of these products. Unlike other nanocarriers, the fabrication of MSNs is a simple and cost-effective process. Moreover, these MSNs have an additional scope of being a multifunctional nanocarrier for spatial, temporal placement of drugs and also for theranostic purpose and imaging, and also supports multidrug loading. Remarkable outcomes have been achieved in this regard in both cellular and preclinical studies. However, certain challenges lay ahead in the successful translation of this platform to bedside. Synthesis of MSNs with consistent characteristics and quality can be a major challenge. The industrial transfer of technology mainly depends on scalability and hence the synthesis of MSNs at production scale may be a barrier to its commercialization. There is a need for a better understanding and control of the manufacturing process to ensure reproducibility in the product. In addition, all drugs cannot be loaded in the same concentration and hence the amount of MSN may vary from case to case which may play a role in determining the maximum tolerated dose of MSN. Certain process analytical tools such as custom-built fluorescence correlation spectroscopy (FCS) coupled with size exclusion/gel permeation chromatography (GPC) adopted by Chen et al. [291] would aid in monitoring the particle size and long term stability and thus reduce batch-to-batch variation. While the inherent toxicity issues of most of the inorganic nanoparticles remains a major issue, encouraging reports on the efficacy and biocompatibility of MSNs in animal models shows the tremendous potential of shifting this platform to clinical levels. However, the difference in the physiology of small animals and humans may lead to failure of these carriers in clinical trials. Lack of in-depth understanding of the interaction between MSNs and the biological system needs to be addressed. Comprehensive in vitro screening assays with varying ligands to ensure optimum uptake, stability, specificity and pharmacokinetic profile would be useful in developing a more reliable product for clinical trials especially for anticancer therapy and the same could be extended as a guide to develop more reliable MSN products for other biomedical purposes as well. Recently, a ray of light for the use of silica nanoparticles was seen in the form of FDA’s approval to conduct stage I human clinical trial for Cornell dots (C dots). This marked an important step towards the acceptance of silica nanoparticles. Following this, first-in human studies by Bukara and group [235] demonstrated the safety of MSNs. Nevertheless, the potential challenge to the clinical translation of MSN-based drug delivery system lies in the lack of substantial evidence on its chronic toxicity studies, genotoxicity and teratogenic potential, long-term tissue compatibility. Thorough understanding of the degradation mechanism of mesoporous silica in vivo is yet to be established. Efforts are to be made by researchers like us to bridge the gap between the preclinical and clinical use of MSNs to achieve marked progress in this subject. We anticipate that if a careful assessment during the production and in vitro evaluation along with studies to ascertain the biosafety of MSNs is performed, these novel designs can be a vital breakthrough in the future for clinical applications in the diagnosis, imaging and treatment catering to the needs of patients.

## Figures and Tables

**Table 1:** List of some marketed products containing nanoparticles.

Marketed Product	Formulation	Drug	Use	References
AmBisome^®^	Liposome	Amphotericin B	Antifungal	[13]
DaunoXome^®^	Liposome	Daunorubicin	Kaposi’s sarcoma associated with HIV	[14]
Doxil^®^	Liposome	Doxorubicin	Kaposi’s sarcoma associated with HIV, breast cancer, ovarian cancer	[15]
Myocet^®^	Liposome	Doxorubicin	Breast cancer	[16]
Emend^®^	Nanocrystals	Aprepitant	Antiemetic	[17]
Megace ES^®^	Nanocrystals	Megestrol acetate	Anorexia	[17]
Tricor^®^	Nanocrystals	Fenofibrate	In hypercholesterolemia	[17]

**Table 2:** List of some of the types of mesoporous silica nanoparticles (MSNs) and their characteristics.

MSN Family	MSN Type	Pore Symmetry	Pore Size (nm)	Pore Volume (cm^3^/g)	References
M41S	MCM-41	2D hexagonal *P*6*mm*	1.5–8	>1.0	[42,43]
	MCM-48	3D cubic I*a*3*d*	2–5	>1.0	[42,43]
	MCM-50	Lamellar *p*2	2–5	>1.0	[44,45]
SBA	SBA-11	3D cubic *Pm*3*m*	2.1–3.6	0.68	[45,46,47]
	SBA-12	3D hexagonal *P*6_3_/*mmc*	3.1	0.83	[48,49,50]
	SBA-15	2D hexagonal *p*6*mm*	6–0	1.17	[43,51]
	SBA-16	Cubic I*m*3*m*	5–15	0.91	[43,52]
KIT	KIT-5	Cubic F*m*3*m*	9.3	0.45	[53,54]
COK	COK-12	Hexagonal *P*6*m*	5.8	0.45	[55,56]

MCM-Mobil Crystalline Materials; SBA- Santa Barbara Amorphous; KIT- Korea Advanced Institute of Science and Technology, COK- Centre for Research Chemistry and Catalysis.

**Table 3:** List of commonly used chemicals in the synthesis of MSNs.

Chemical Constituents	Function	References
Cetyltrimethylammonium bromide (CTAB)	Structure directing agent/template	[94,95]
Cetyltrimethylammonium chloride (CTAC)	Structure directing agent/template	[96,97]
Pluronic F123, F127	Surfactant template	[38,98]
Brij-76	Surfactant template	[99,100]
Triton X-100	Surfactant	[101,102]
Tween 20, 40, 60, 80	Surfactant	[103]
Tetraethyl orthosilicate (TEOS)	Inorganic silica source	[94,95]
Tetramethoxy silane (TMOS)	Inorganic silica source	[104,105]
Tetrakis(2-hydroxyethyl) orthosilicate (THEOS)	Inorganic silica source	[106]
Trimethoxyvinylsilane (TMVS)	Inorganic silica source	[107]
Sodium silicate	Inorganic silica source	[108]
Ethanol	Cosolvent to solubilize TEOS	[97,109]
Sodium hydroxide (NaOH)	Base catalyst	[95]
Ammonium hydroxide (NH_4_OH)	Base catalyst	[94]
Triethanolamine (TEA)	Base catalyst, complexing agent and growth inhibitor	[96]
Diethanolamine (DEA)	Base catalyst	[96,109]
Disodium hydrogen phosphate-sodium dihydrogen phosphate buffer solution	Reaction medium	[109]
Triisopropylbenzene (TIPB)	Pore-expanding agent	[110,111]
Tetrapropoxysilane (TPOS)	Pore- expanding agent	[111]
Pluronic polymer P103	Pore-expanding agent	[112]

**Table 4:** Comparison of loading in MSNs.

Carrier	Drug	Loading (wt %)	References
MCM-41	Ibuprofen	35.9	[75]
HMSNs		74.5	
MCM-41	Doxorubicin	48.16	[74]
HMSNs		112.12	
HMSNs	5-fluorouracil	18.54	[78]
HMSNs-NH_2_		28.89	
HMSNs-COOH		20.73	
HMSNs-CN		22.54	
HMSNs-CH_3_		12.13	
MCM-41_(C12)_	Captopril	23.6	[151]
MCM-41_(C16)_		34	
SBA-15		22.6	
MCM-41	Erythromycin	29	[152]
SBA-15		34	
SBA-15 (C8)		13	
SBA-15 (C18)		18	
MCM-41	Alendronate	14	[153]
MCM-41-NH_2_		37	
SBA-15		8	
SBA-15-NH_2_		22	
MSN-C0	Lysozyme	34	[149]
MSN-C10		42	

HMSNs—Hollow mesoporous silica nanoparticles.

**Table 5:** Comparison of release rate of MSNs.

Carrier	Drug	Release Rate	References
MCM-41 (C12)	Captopril	45 wt % within 2 h, total drug release over 16 h	[151]
MCM-41 (C16)		47.47 wt % within 2 h, total drug release >30 h	
SBA-15		60 wt % within 0.5 h, total drug release over 16 h	
SBA-15	Erythromycin	60% release within 5 h, total drug release within 14 h	[152]
SBA-15 (C8)			
SBA-15 (C18)			
SBA-15 unmodified (PS0)	Ibuprofen	Complete release in 10 h	[156]
SBA-15-NH_2_ by post synthesis (PS2)		Initial burst release of 50% in 10 h followed by 100% release in 3 days	
SBA-15-NH_2_ by one pot synthesis (OPS2)		Complete release in 10 h	
MSN (grafting-loading approach)	Doxorubicin	40% in 8 h and stagnant release beyond 8 h	[143]
MSN (loading-grafting approach)		10% in first 24 h, sustained beyond 160 h	

**Table 6:** List of some biomedical applications of MSNs.

Category	Drug	Carrier	PS, D_p_ (nm)	Activity Testing	References
Anticancer	Doxorubicin	Hollow MSNs	120, 2.7	HeLa cells	[157]
		Lipid-coated MSNs	295, 2.3	MCF-7 human breast cancer cells	[158]
	Topotecan	MCM-41	72.9, 2.7	Female athymic nude mice injected with MDA-MB-231 human epithelial breast cancer cells s.c.	[159]
	Quercetin	MCM-41			[159]
	Curcumin	SLN-silica microcapsules	305, 7.8	Caco-2 cells	[160]
	Paclitaxel	MSNs	100, 2.3	PK studies in peritoneal MIA-PaCa-2 (human pancreatic cancer cell) tumour bearing nude mice	[161]
	5-fluorouracil	MCM-41	135, 2.9	Human colonic HT-29 cells	[162]
	Etoposide	MCM-41-PAA	142.85, 3.69	PC-3 and LNCaP human prostate cancer cells	[163]
	16-hydroxy-cleroda-3,13-dien-16,15-olide (HCD)	Eudragit S100-HCD-Cu-MSN	514, 4.3	Athymic male nude mice injected with C6 Glioma cells s.c.	[164]
Antidepressant	Duloxetine hydrochloride	MSNs	PS not reported, 2.6–7.2	Not reported	[165]
Anti-tuberculosis	Rifampicin	MCM-41	218, 2.4	Not reported	[166]
Anti-inflammatory	Ibuprofen	MSNs	PS not reported, 3.6–4.1	Not reported	[101]
	Ketoprofen	MCM-41 SBA-15	1500, 3.4 970, 6.24	Not reported	[167]
	Budesonide	MCM-41	100, 2.7	Coculture of HT-29 cells and PMA treated human EOL-1 (acute myeloid eosinophilic leukemia) cells	[168]
Antibacterial	Ciprofloxacin	Lipid-coated MSNs	80–100, D_p_ not reported	*Salmonella typhimurium* administered mice	[169]
	Ciprofloxacin	Arg-MSN	75, 7.2	*Salmonella* infected BALB/*c* mice	[170]
	Tetracycline	MCM-41	41 and 406, D_p_ not reported	In vitro against *Escherichia coli*	[171]
Antihypertensive	Captopril	SBA-15	PS not reported, 7.15	Not reported	[172]
	Aliskiren	SBA-15			[172]
	Hydrochlorothiazide, Losartan potassium, Amlodipine besylate, Simvastatin	MCM-41 polypill	150, 4.64	Not reported	[173]
Hypoglycemic drugs	Gluconated insulin Rosiglitazone maleate	Alizarin complexone-MSNs	60–100, ~2.3	In vitro monitoring of drug release in human serum	[174]
	16-hydroxycleroda-3,13-dine-16,15-olide (HCD)	MSNs	259, 3.9	Diet-induced ICR male diabetic mice	[175]
Osteogenic	Alendronate	MCM-41 SBA-15	PS not reported, 3.8 PS not reported, 9.0	Not reported	[153]
	Alendronate	HA-AL-MS-PLGA microspheres	245–258 µm	In vitro on synovium-derived mesenchymal stem cells (MSCs)	[176]
	Dexamethasone	MCM-41	265, D_p_ not reported	Male Sprague−Dawley rats	[177]
Antioxidant	Morin	MSNs	150, 3	In vitro	[178]

PS—Particle size; D_p_—Pore diameter; SLN—Solid lipid nanoparticles; HA-AL-MS-PLGA-Hydroxyapatite-Alendronate-Mesoporous silica-poly(lactic-co-glycolic acid); Arg-MSN—Arginine coated MSN; MCF- Michigan Cancer Foundation; MDA-MB- M.D. Anderson- Metastasis breast cancer; MIA- Melanoma inhibitory activity; PK—Pharmacokinetics; PMA—phorbol myristate acetate ester; PAA—Polyacrylic acid.

**Table 7:** List of some functionalized MSNs explored for tumour targeting.

Drugs	Application	Targeting Ligand	Receptor	References
5-fluorouracil	Colorectal cancer	Hyaluronic acid	CD44	[189]
5-fluorouracil	Colorectal cancer	EGF	EGF	[190]
Curcumin	Cervical cancer	Chondroitin sulphate	CD44	[191]
Docetaxel	Breast cancer	Folic acid	Folate	[181]
Docetaxel	Hepatoma	Lactose	Asialoglycoprotein	[184]
Doxorubicin	Hepatic cancer	Transferrin	Transferrin	[192]
Doxorubicin	Colon cancer	Aptamer	(EpCAM)	[193]
Photosensitizer merocyanine	Breast cancer	Mannose	Mannose	[194]
Quercetin	Triple negative breast cancer	cRGD peptide	Integrin receptor αvβ3	[159]
Quercetin	Breast cancer	Folic acid	Folate	[195]
Sunitinib	Glioblastoma	VEGF_121_	VEGF	[196]
Topotecan	Triple-negative breast cancer	cRGD peptide	Integrin receptor αvβ3	[159]

EGF—Epidermal growth factor; VEGF—Vascular endothelial growth factor; EpCAM—Epithelial cell adhesion molecule.
